# Diversity patterns, abiotic and biotic drivers, and future dynamics of native invasive plants on the Qinghai-Tibet Plateau

**DOI:** 10.3389/fpls.2025.1715360

**Published:** 2025-11-13

**Authors:** Wanyin Xiong, Tao Cheng, Shijian Liu, Xin Liu, Hechun Ding, Mengdie Yin, Wenguang Sun, Yazhou Zhang

**Affiliations:** 1 School of Life Sciences, Yunnan Normal University, Kunming, Yunnan, China; 2 Yunnan Key Laboratory of Plant Diversity and Biogeography, Kunming Institute of Botany, Chinese Academy of Sciences, Kunming, Yunnan, China; 3 Southwest United Graduate School, Kunming, China; 4 School of Ecology and Environment, Southwest Forestry University, Kunming, China

**Keywords:** native invasive plants, human activities, climate change, allelochemical diversity, biological invasion

## Abstract

**Introduction:**

Compared to alien invasive plants, native invasive plants have long been overlooked. As a result, many biodiversity hotspots are threatened by invasions of native species, yet lack sufficient policy attention and management interventions. This study focuses on native invasive plants on the Qinghai-Tibet Plateau (QTP) as a case study, aiming to provide guidance for regional management and offer insights for related research in other areas.

**Methods:**

We compiled a comprehensive dataset of 83 native invasive plants and environmental drivers on the QTP. Using spatial statistics and ensemble modeling, we analyzed invasion patterns and projected future trends.

**Results:**

A distinct northwest-to-southeast richness gradient was found, with the southeast as the primary invasion hotspot. This pattern aligned closely with allelochemical diversity, primarily benzenoids, terpenoids, and flavonoids. Invasion distribution was jointly influenced by allelochemicals, human activities, and climate. Models projected intensification and northwestward expansion of hotspots, increasing risks to protected areas, with invasive hotspot areas expanding by approximately 178.8×10^4^ km^2^ across scenarios. Moreover, the MaxEnt model demonstrated extremely high predictive accuracy, with the average test AUC for all species reaching 0.9834.

**Discussion:**

We propose targeted management focusing on the southeastern QTP, including allelochemical monitoring via metabolomics and biocontrol using allelopathy-resistant forage grasses and compound-degrading microbes to improve conservation efficiency and adaptability. Our findings unravel the large-scale mechanisms of alpine plant invasions while translating theoretical advances into practical management strategies for this ecologically critical landscape.

## 1 Introduction

Biological invasions continue to escalate as a major ecosystem challenge, with invasive alien species remaining a central focus of scientific and policy efforts (Pyšek et al., 2020). Alien species, defined as organisms introduced beyond their natural ranges and often across geopolitical borders, have been widely documented to cause severe biodiversity loss and substantial economic damage (Diagne et al., 2022). However, traditional frameworks of alien invasion overemphasize biogeographic origins, thereby excluding many impactful invasion damages from current governance systems, including severe ecological threats already posed by overlooked native invasive plants (Valéry et al., 2008). Native invasive species refer to indigenous organisms that, when human-mediated into new regions within their native range, exhibit invasive characteristics, causing significant disruption to local flora, ecosystem functioning, and local economies comparable to that of alien invaders (Carey et al., 2012; Pivello et al., 2018). A plant’s invasive status should be determined by its ecological impact rather than its origin (Carey et al., 2012; Pivello et al., 2018). Therefore, native plant invasion is specifically defined as the expansion of indigenous species causing demonstrated ecological damage, excluding those merely forming transient communities or representing successional stages without measurable negative impacts. Critical knowledge gaps remain concerning the spatial distribution patterns, key drivers (including emerging abiotic and biotic determinants), and future dynamics of native invasive plants. Addressing these gaps is urgently needed, as such understanding will elucidate the mechanisms behind native invasive plants and inform targeted management strategies for vulnerable ecosystems, e.g., high-elevation regions.

Biological invasions are driven by the synergistic interaction of climatic, anthropogenic, and allelochemical factors that collectively reshape invasion patterns and ecosystem impacts. Climate change acts as a selective filter that differentially influences non-invasive and invasive plants (Hellmann et al., 2008; Hulme, 2016), with invasive plants exhibiting superior adaptation to extreme climatic factors, particularly temperature (Sharma et al., 2023). Under warming scenarios, invasive plants display enhanced phenological plasticity, increased biomass production, stronger allelopathic effects, and improved overwintering capacity that collectively facilitate their expansion into higher latitudes and elevations (Saqib et al., 2025). This climate-mediated polarization imposes novel ecological stresses through direct climatic challenges and intensified competition (Chen et al., 2024), potentially leading to biodiversity loss, simplified ecological networks, and degraded ecosystem functions (Zhao et al., 2021). Understanding these relationships is crucial for predicting native invasive plant spread (Thuiller et al., 2007) as changing climate regimes alter distribution boundaries and competitive interactions within native ranges (Hellmann et al., 2008).

Human activities have become the dominant invasion driver in the Anthropocene, often exerting more immediate effects than climate change itself (Hulme, 2009). As primary contributors to the sixth mass extinction (Ceballos et al., 2015), human-mediated disturbances facilitate invasions through global trade networks that transport propagules, infrastructure development that fragments habitats, and economic activities that alter biomes. Empirical studies demonstrate strong correlations between invasion hotspots and human pressure indicators like population density, economic outputs, and transportation infrastructures (Hulme, 2009). Linear infrastructure projects particularly create invasion corridors while causing soil disturbance and habitat fragmentation (Deng et al., 2020), often acting synergistically with climate change to accelerate ecosystem transformation (Oliver and Morecroft, 2014).

Although underexplored in large-scale invasion ecology (Qin et al., 2024), allelochemical diversity offer crucial mechanistic insights via the Novel Weapons Hypothesis (Callaway and Ridenour, 2004). This hypothesis posits that invasive plants succeed by employing unique allelopathic compounds that native plants and soil biota cannot counteract (Hierro and Callaway, 2003). These phytochemicals alter community dynamics by inhibiting key processes such as seed germination, seedling establishment, and microbial interactions (Inderjit and Duke, 2003; Karban, 2007). This mechanism is demonstrated by the native invasive plants *Ligularia cymbulifera*, whose potent allelochemicals suppress forage species recruitment and contribute to grassland degradation (Wang et al., 2022). Despite evidence of allelopathy’s local-scale influence on invasion success (Inderjit et al., 2011), its macroecological consequences remain poorly understood. In general, synergistic interactions among these drivers create amplification loops: climate warming enhances allelochemical production and efficacy; human activities disperse chemically armed propagules while creating disturbed habitats more susceptible to invasion, and habitat fragmentation increases native community vulnerability to biochemical disruption. This integrated framework emphasizes that effective management strategies must address these interconnected mechanisms, particularly for emerging threats like native invasive plants in rapidly changing environments.

The Qinghai-Tibet Plateau (QTP), often referred to as the “Roof of the World” and “Asia’s Water Tower”, hosts the world’s most diverse temperate flora and a high concentration of endemic species (Yu et al., 2019). This unique region is experiencing particularly rapid climate change, with warming rates exceeding the global average (Yao et al., 2019), which triggers cascading environmental impacts including glacial retreat, permafrost degradation, and pasture desertification (Zhang et al., 2025). The QTP is experiencing rapid transformations coupled with growing human pressures (Zhao et al., 2022); these factors collectively make it an ideal natural laboratory for investigating invasion mechanisms. Our work specifically examines the synergistic effects of climatic, anthropogenic, and allelochemical drivers under accelerated global change. The plateau’s sharp environmental gradients, well-documented flora, and clearly delineated human disturbance gradients provide an unparalleled context for disentangling the complex interactions highlighted in our conceptual framework. Plant diversity on the QTP represents not only a critical ecological asset but also a fundamental pillar of China’s national ecological security and sustainable development (Zhang et al., 2018). However, the aggressive spread of native invasive species threatens these values by diminishing biodiversity, altering resource-use efficiency through competitive niche occupation, and compromising agroforestry systems and the local economy (Wang et al., 2022). Despite these threats, critical knowledge gaps persist regarding how the key drivers identified in our framework, climatic filters, human disturbances, and allelochemical weapons, interact to shape invasion patterns across this sensitive region. This gap is particularly concerning given the QTP’s ecological vulnerability and strategic importance.

To address these pressing questions, we employ an integrative analytical framework to investigate native invasive plants across QTP. This framework synthesizes three key drivers derived from the preceding literature: climatic influences on species distributions, human-mediated dispersal and disturbance, and chemically mediated biotic interactions. Accordingly, we specifically examine the roles of climatic variables, human factors (e.g., population density, road networks, and economic indicators), and plant-specific traits including allelochemical diversity. Our study is therefore guided by three corresponding research questions: (1) What biogeographic patterns characterize the distribution of native invasive plants across the QTP’s environmental gradients? (2) Which combination of climatic, anthropogenic, and biological factors most strongly drives these invasion patterns? (3) How are invasion hotspots projected to shift under future climate change scenarios? By addressing these questions, we aim to provide mechanistic insights into alpine plant invasions and advance the theoretical understanding of high-elevation invasion ecology. Furthermore, we seek to deliver practical recommendations for managing these species in this ecologically sensitive and strategically important landscape.

## 2 Materials and methods

### 2.1 Data collection

This study focuses on the Qinghai-Tibet Plateau (QTP) of China, encompassing the Xizang Autonomous Region, northwestern Yunnan, western Sichuan, Qinghai, southern Xinjiang, and southwestern Gansu provinces (Zhang et al., 2016). The region is characterized by highly heterogeneous topographic features, including deep canyons, high mountain ranges, rolling hills, and broad plateaus. It also supports various vegetation types, such as valley shrubs, broadleaf and coniferous forests, alpine shrubs, meadows, and subnival belts (Zhang et al., 2021b). We conducted a systematic literature review to identify all published research that investigated native invasive plant species and their allelochemicals within the QTP region. We classified a plant as a native invasive plant if it met all three criteria: natural occurrence within the Qinghai-Tibet Plateau, ability to form hyper-dominant communities, and well-documented evidence of negative ecological impacts. A checklist of native invasive plants (total 83 species) was compiled through systematic searches of scientific databases on 29 Oct 2024. The searched databases included China National Knowledge Infrastructure (https://c61.oversea.cnki.net/), Web of Science (https://www.webofscience.com/wos/), and Springer (https://link.springer.com/). The complete checklist is provided in **Supplementary Table S1**. Allelochemicals reported in the literature were classified into 15 categories (**Supplementary Table S1**): (1) benzenoids, (2) terpenoids, (3) flavonoids, (4) organic nitrogen compounds, (5) alkaloids, (6) organic heterocycles, (7) phenols, (8) nucleosides, (9) organic acids, (10) shikimates and phenylpropanoids, (11) carbohydrates, (12) lipids and lipid-like molecules, (13) fatty acids, (14) lignans, and (15) other compounds (remaining unclassified compounds).

To map the spatial distribution of these native invasive plants, we compiled species-level occurrence data from national and regional floras, including *Flora Reipublicae Popularis Sinicae* (Editorial Committee of Flora of, 1961–2002); *Flora of China* (Lin et al., 2013); *Flora Yunnanica* (Kunming Institute of Botany, Chinese Academy of Sciences, 2006); *Flora Xizangica* (Kunming Institute of Botany, Chinese Academy of Sciences, 1983); *Flora Xinjiangensis* (Xinjiang Flora Editorial, 1993–1996); *Flora Gansuensis* (Gansu Flora Editorial, C, 2005); and *Flora Sichuanica* (Sichuan Flora Editorial, 1981–2007). Additional occurrence records were sourced from the Chinese Virtual Herbarium (https://www.cvh.ac.cn/); the Herbarium of the Kunming Institute of Botany, Chinese Academy of Sciences (http://groups.kib.cas.cn/kun/); and the Global Biodiversity Information Facility (https://www.gbif.org/). Socioeconomic data, including population and gross domestic product (GDP), were obtained from the National Bureau of Statistics of China (http://www.stats.gov.cn/). Spatial data on road density were extracted from the Geographic Data Platform of Peking University’s College of Urban and Environmental Sciences (http://geodata.pku.edu.cn/). These data were collected until Dec 2024.

### 2.2 Spatial analysis

Species distribution data were processed following a standardized data-cleaning protocol established by Zhang (Zhang et al., 2021a; Zhao et al., 2022), involving a three-tier quality control workflow: (1) For each target species, distribution records were compiled from multiple authoritative sources, including herbarium specimens, type localities from regional floras, and peer-reviewed habitat descriptions, to establish a high-confidence baseline database for subsequent validation and calibration. (2) Verified records were systematically evaluated to identify and correct biologically implausible occurrences and spatially biased coordinates, ensuring consistency. To mitigate spatial sampling bias, we applied a spatial thinning procedure using the “CoordinateCleaner” package (Zizka et al., 2019) to reduce record clustering in easily accessible areas. (3) Geocoding techniques were applied to convert descriptive location information (e.g., village names, mountain ranges) into standardized geographic coordinates. Given the vast average area of county-level administrative units on the QTP (~14,900 km², with the largest exceeding 200,000 km²), county-level records lacking precise coordinates were georeferenced to 0.5° × 0.5° grid cells. This resolution has been validated as an effective balance between spatial accuracy and data coverage in alpine ecosystems (Yu et al., 2019; Zhang et al., 2022). The georeferencing procedure involved (a) dividing all county units into 0.5° grids; (b) extracting elevation ranges (maximum and minimum) for each grid; (c) filtering grids based on species-specific elevation preferences (Zhang et al., 2021b); and (d) removing duplicate species records by retaining only a single occurrence per species within each 0.5° grid cell. This elevation-filtering approach has been successfully employed in multiple recent studies focusing on the Qinghai-Tibet Plateau region (Zhang et al., 2021b; Zhao et al., 2022) and has been shown to significantly improve distribution accuracy when precise coordinate data are unavailable. Road density was calculated by summing the total length of national highways and provincial and county roads within each grid cell and dividing by the grid area. Population density and GDP data were similarly processed, with all socioeconomic variables interpolated across the plateau using spatial kriging methods (Wang et al., 2010) and aggregated to the grid levels. Climate variables (Bio1-19) were extracted from the WorldClim v.2.1 database (Fick and Hijmans, 2017), with mean values computed for each grid (**Table 1**). The spatial resolution of these data is uniformly 2.5 arc minutes (~4.5 kilometers), and they are registered in the WGS_1984 geographic coordinate system. Allelochemical diversity per grid was quantified as the number of distinct allelochemicals associated with invasive species present within each grid cell. All spatial analyses were performed using ArcGIS 10.4 (http://www.esri.com).

**Table 1:** Definitions of different climate variables.

Climate variable	Definition
Bio1	Annual mean temperature
Bio2	Mean diurnal range
Bio3	Isothermality
Bio4	Temperature seasonality
Bio5	Max temperature of warmest month
Bio6	Min temperature of coldest month
Bio7	Temperature annual range
Bio8	Mean temperature of wettest quarter
Bio9	Mean temperature of driest quarter
Bio10	Mean temperature of warmest quarter
Bio11	Mean temperature of coldest quarter
Bio12	Annual precipitation
Bio13	Precipitation of wettest month
Bio14	Precipitation of driest month
Bio15	Precipitation seasonality
Bio16	Precipitation of wettest quarter
Bio17	Precipitation of driest quarter
Bio18	Precipitation of warmest quarter
Bio19	Precipitation of coldest quarter

### 2.3 Statistical analysis

The richness of native invasive plant species was calculated based on the number of species within each grid cell. A phylogenetic tree was constructed using the “V.Phylomaker” package (Jin and Qian, 2019) to illustrate evolutionary relationships among native invasive plant species based on the compiled species checklist. Heatmaps were generated to visualize the distribution of allelochemical diversity across taxa to complement the tree. Random forest model was used as an initial screening tool to identify the most important predictors of native invasive plant distribution from our high-dimensional environmental dataset, while handling nonlinear relationships and interactions without prior assumptions. We first employed random forest models using the “randomForest” (Liaw and Wiener, 2002) and “rfPermute” (Archer, 2023) packages in R (v4.4.1) (R Core Team, 2024). The random forest algorithm is well-suited for high-dimensional ecological data, as it inherently mitigates predictor collinearity by averaging across numerous decision trees (Yang et al., 2021). Variable importance was computed using the permutation-based method. We built a forest of 500 trees, performing 1000 permutations per variable to assess importance. The metric used was the %IncMSE (percentage increase in mean squared error), which quantifies the decrease in prediction accuracy when a variable’s values are randomly shuffled in out-of-bag samples. The resulting importance values were scaled to facilitate comparison across predictors, and statistical significance was evaluated using permutation-derived p-values. As tree-based algorithms like random forest are insensitive to the scale of predictor variables, we did not apply feature scaling prior to model training. From the ranked variable importance output, we selected the top seven drivers for subsequent model fitting: allelochemicals, GDP, road, Bio15 (precipitation seasonality), population, Bio18 (precipitation of warmest quarter) and Bio3 (isothermality).

Ordinary least squares (OLS), mixed-effect, and spatial autoregressive models (SAR) models were employed as complementary approaches to assess the direction and strength of relationships between key drivers and invasion patterns. OLS and mixed-effect models provided a baseline understanding, while SAR explicitly accounted for spatial autocorrelation, ensuring robust inference. We fitted OLS to analyze the relationships between species richness and the selected predictors, including human factors, climate variables, and allelochemicals. We further fitted linear mixed-effects models using the “lme4” (Bates et al., 2015) package. In these models, the selected drivers were treated as fixed effects, while grid cells were included as random effects to account for spatially structured variance and to improve the estimation of fixed-effect coefficients. We performed Moran’s I test to examine spatial autocorrelation in the input variables. Significant spatial clustering was observed across key variables, particularly population density, GDP, and road network density, reflecting the inherent spatial nature of these indicators. To address the spatial autocorrelation, we refitted the linear models using spatial autoregressive models (SAR), allowing for spatial dependencies in the error terms. A spatial error model (SARerror) implemented using the “spatialreg” package (Bivand and Piras, 2015), which accounts for spatial dependence in the regression residuals. Constructed using k-nearest neighbors with k=4 neighbors based on Euclidean distances between sampling locations. This step ensured a more accurate coefficient estimate and enabled a comprehensive comparison of model outcomes across model frameworks. Bayesian mixed-effects models were applied to quantify uncertainty and incorporate hierarchical structure in our data, allowing us to partition variance among climatic, human, and allelochemical factors while providing probabilistic estimates of effect sizes. We conducted Bayesian mixed-effects models using the “brms” package (Bürkner, 2021).

Structural equation models (SEM) were used to test our conceptual framework of direct and indirect pathways through which climatic, human, and allelochemical factors jointly influence invasion success, thereby moving beyond correlation to evaluate causal hypotheses. Based on the results from the OLS and the SAR models, we found an interesting outcome: only climate factors consistently and significantly influenced the diversity of invasive plants in both models. The effects of human activities and allelochemical diversity on invasive plant diversity were not significant after accounting for spatial autocorrelation, yet both were highly overlapped spatially with plant diversity. This indicates that the relationship between human activities, allelochemical diversity, and invasive plant diversity is regulated by other factors (i.e., climate). Here, we used the “piecewiseSEM” package (Lefcheck, 2016) to fit SEM to explore the direct or indirect relationships of human activities and allelochemical diversity, regulated by climatic factors, with the diversity of invasive plants.

### 2.4 Future plant diversity dynamics simulation

Maximum Entropy algorithm (MaxEnt) was implemented to project future distribution shifts under climate change scenarios, leveraging its strengths in presence-only data and transferability to novel environments. Due to the unavailability of future data on human activities and allelochemical distributions, we relied exclusively on projected climate data to predict the dynamics of native invasive plants. Bioclimatic data were obtained for three periods: baseline climate (1970–2000), the near future (2021–2040), and the distant future (2081–2100). Future climate projections were derived from global climate models included in the Coupled Model Intercomparison Project Phase 6, using two Shared Socioeconomic Pathways (SSPs): SSP1-2.6, representing a sustainable development pathway, and SSP5-8.5, representing a high carbon emissions scenario (O’Neill et al., 2016). To reduce multicollinearity among environmental variables, Pearson correlation coefficients were calculated. Variables with correlation coefficients below 0.8 were retained, while among highly correlated variables (r>0.8), the one with the highest contribution was kept, and the variables with minimal (i.e., close to zero) contribution were excluded (Chai et al., 2025).

Species distribution models were constructed using the Maximum Entropy algorithm (MaxEnt version 3.4.4) (Phillips and Dudík, 2008). Model parameters were configured as follows: 10 bootstrap replicates, 10,000 background points, a 75:25 random split for training and validation datasets, and a maximum of 5,000 iterations with a convergence threshold of 0.00001 (Valavi et al., 2021). Feature combinations and regularization multipliers were optimized through batch scripts to improve model generalization. Model performance was evaluated using the Area Under the Curve (AUC), with values >0.9 interpreted as indicating high predictive reliability. With an overall average training AUC of 0.9867 and an average test AUC of 0.9834 for all species, both significantly greater than 0.9 and closely matched, the models demonstrated not only high predictive accuracy but also exceptional stability (**Supplementary Table S2**). The resulting habitat suitability outputs (probability range: 0–1) were converted into binary raster data using a threshold of 0.5, classifying the habitat as either suitable (1) or unsuitable (0) (Gong et al., 2022). Changes in future habitat suitability were determined by subtracting current raster values from future scenario values: positive changes were interpreted as “expanding species,” and negative changes as “contracting species”. To assess spatial patterns of expansion, binary rasters of all expanding species were overlaid in ArcGIS (version 10.4.1) using the “Sum” function, generating cumulative suitable area layers under each scenario. Official protected area boundaries for the QTP were obtained from the National Specimen Information Infrastructure (NSII, http://www.nsii.org.cn). These vector layers were reprojected to match the coordinate reference system of the habitat rasters (GCS_WGS_1984) and clipped to the boundaries of the QTP. Cumulative suitable area layers were then intersected with protected area polygons. Using the “Zonal Statistics” tool in ArcGIS, the number of suitable pixels within each protected area was calculated for each period and scenario. This allowed for spatial quantification of the extent (in km²) to which protected areas may be impacted by the expansion of invasive plants under future climate conditions.

These methods were applied in a logically ordered sequence: variable selection (Random Forest), relationship testing (OLS, mixed-effect, and SAR models), uncertainty and hierarchical estimation (Bayesian models), pathway analysis (SEM), and spatial forecasting (MaxEnt). Together, they form a cohesive analytical framework that progresses from pattern detection to mechanistic understanding and predictive modeling.

## 3 Results

### 3.1 Spatial, taxonomic, and allelopathic characteristics of native invasive plants

**Figure 1:**
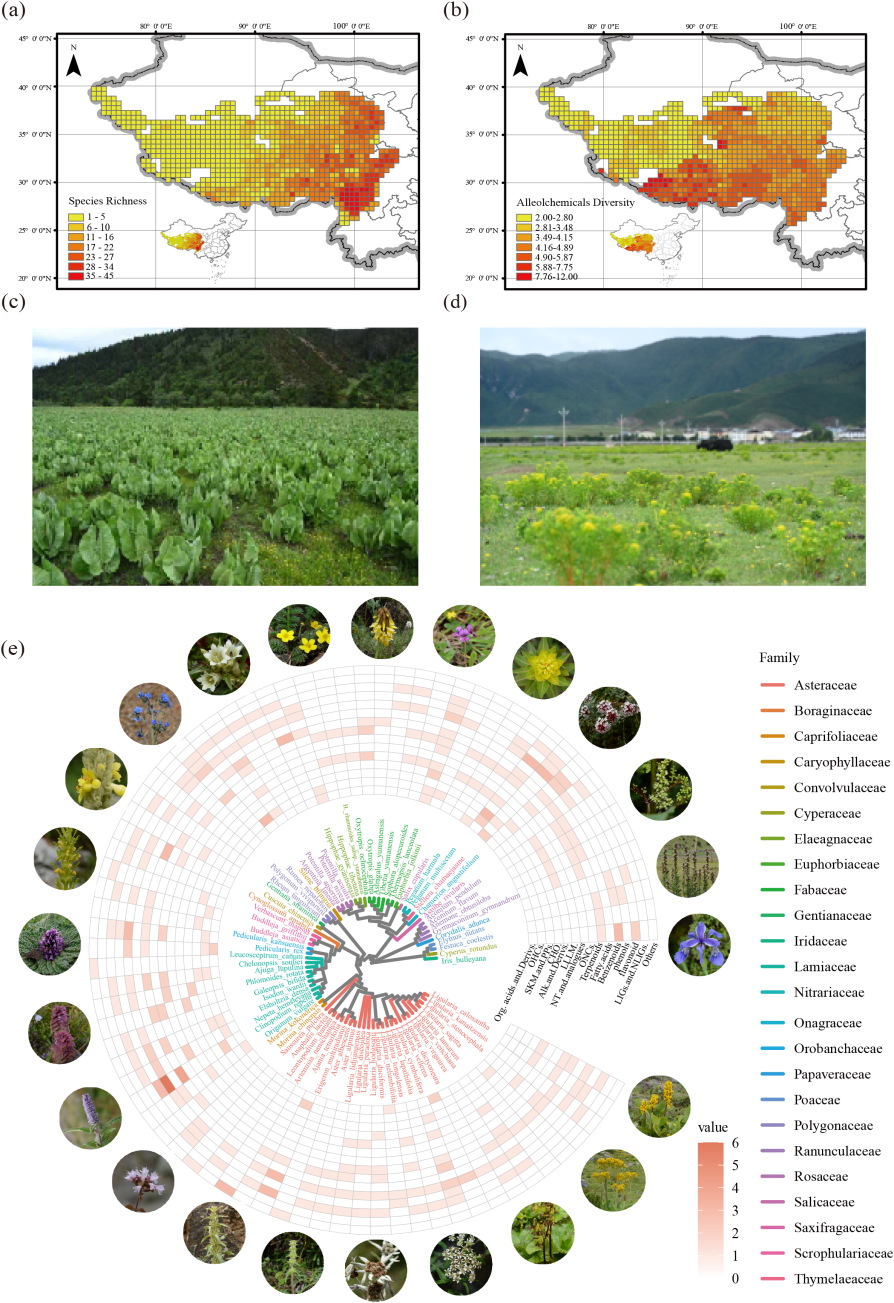
Spatial and phylogenetic patterns of native invasive plant richness and allelochemical diversity across the QTP. **(a)** Spatial distribution of native invasive plant richness. **(b)** Distribution pattern of allelochemical diversity. Community landscapes invaded by *Ligularia cymbulifera***(c)** and *Euphorbia jolkinii***(d)** are shown as representative examples of habitat alternation due to native invasive species. **(e)** A phylogenetic tree and corresponding heatmap show the number and types of allelochemicals across invasive species. Org.acids.and.Derivs stands for Organic acids and derivatives, OHCs stands for Organoheterocyclic compounds, SKM.and.PPs stands for Shikimates and Phenylpropanoids, CHO stands for Carbohydrates, Alk.and.Derivs stands for Alkaloids and derivatives, LLLM. stands for Lipids and lipid-like molecules, NT.and.analogues stands for Nucleosides nucleotides and analogues, ONCs stands for Organic nitrogen compounds, LIGs.and.NLIGs stands for Lignans neolignans.

The species richness of native invasive plants across the QTP increased progressively from the northwestern to southeastern regions, with the southeastern plateau showing a pronounced clustering pattern and hosting the highest concentration of high-richness areas (**Figure 1a**). Lower allelochemical diversity was observed in the northwest. At the same time, higher levels were observed in the southwest and southeast regions of the plateau (**Figure 1b**), indicating a spatial concordance between plant richness and allelopathic potential in these areas. We presented invasion landscapes of two native invasive plant species, *Ligularia cymbulifera* (**Figure1c**) and *Euphorbia jolkinii* (**Figure1d**), to exemplify their detrimental impacts. Taxonomically, 25 families of native invasive plants were identified in the QTP. The Asteraceae family was the most species-rich, contributing 26 species, followed by the Lamiaceae with 10 species and the Fabaceae with 6 species. In terms of allelochemical composition, benzenoids were the most abundant, followed by terpenoids and flavonoids (**Figure 1e**).

### 3.2 Relative importance of abiotic and biotic drivers

**Figure 2:**
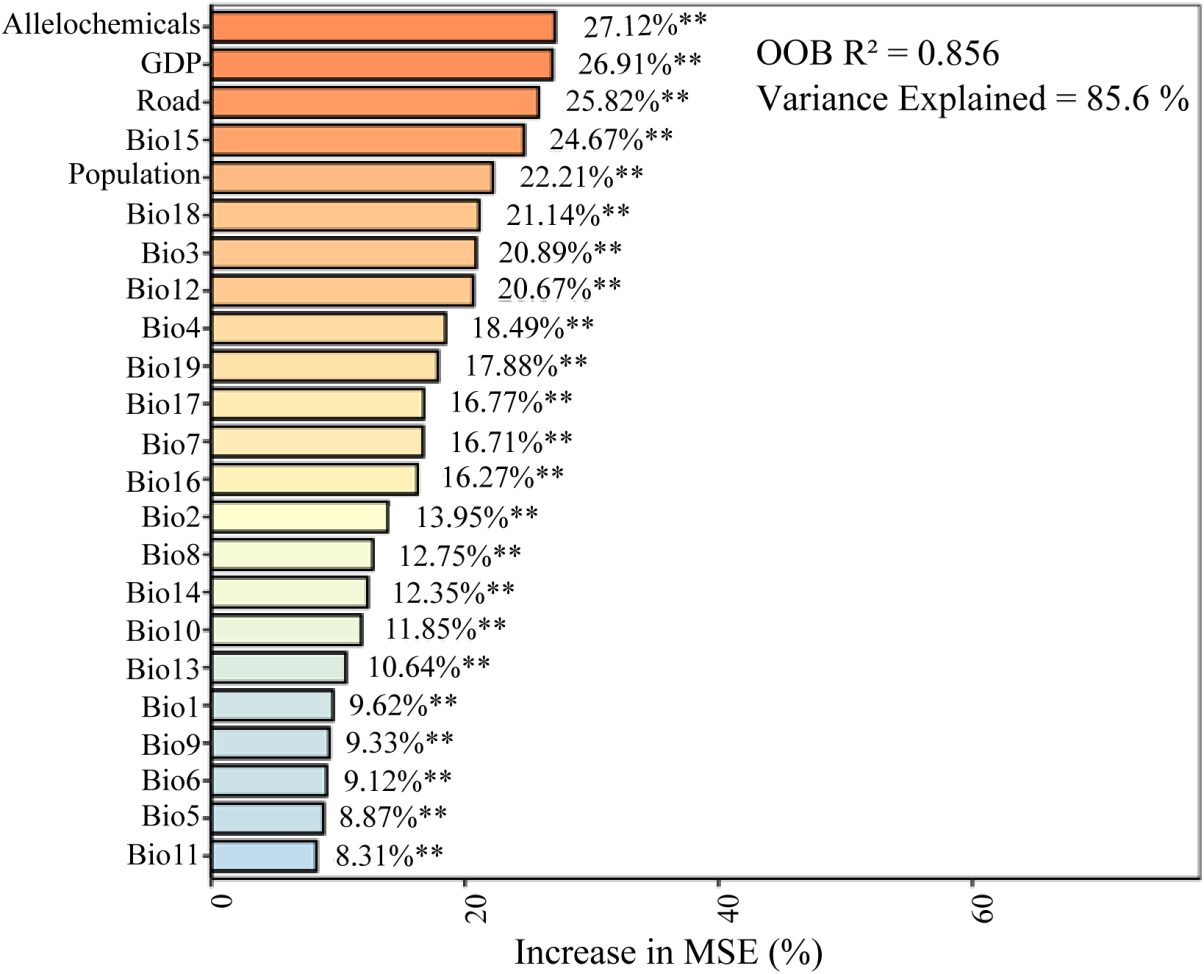
The relative importance of multiple drivers influencing the distribution of native invasive plants on the QTP, as determined by random forest modeling. Significance levels: **P < 0.01. Bio1: annual mean temperature, Bio2: mean diurnal range, Bio3: isothermality, Bio4: temperature seasonality, Bio5: max temperature of warmest month, Bio6: min temperature of coldest month, Bio7: temperature annual range, Bio8: mean temperature of wettest quarter, Bio9: mean temperature of driest quarter, Bio10: mean temperature of warmest quarter, Bio11: mean temperature of coldest quarter, Bio12: annual precipitation, Bio13: precipitation of wettest month, Bio14: precipitation of driest month, Bio15: precipitation seasonality, Bio16: precipitation of wettest quarter, Bio17: precipitation of driest quarter, Bio18: precipitation of warmest quarter, and Bio19: precipitation of coldest quarter.

The Random Forest model demonstrated exceptional predictive performance, explaining 85.6% of the variance in species richness (OOB R^2^ = 0.856), indicating that the selected environmental and anthropogenic variables effectively capture the key drivers governing distribution patterns across the Qinghai-Tibet Plateau. The model identified seven influential predictors: allelochemicals, GDP, road density, Bio15, population density, Bio18, and Bio3 (**Figure 2**). Their high sensitivity was evidenced by 20-40% increases in mean squared error when these variables were permuted. Notably, allelochemicals emerged as the most critical factor, accounting for 27.12% of the total variable importance. These findings collectively highlight the central role of allelochemical diversity in shaping the spatial distribution of native invasive plant species on the QTP, while confirming the robust explanatory power of the integrated predictor set.

**Figure 3:**
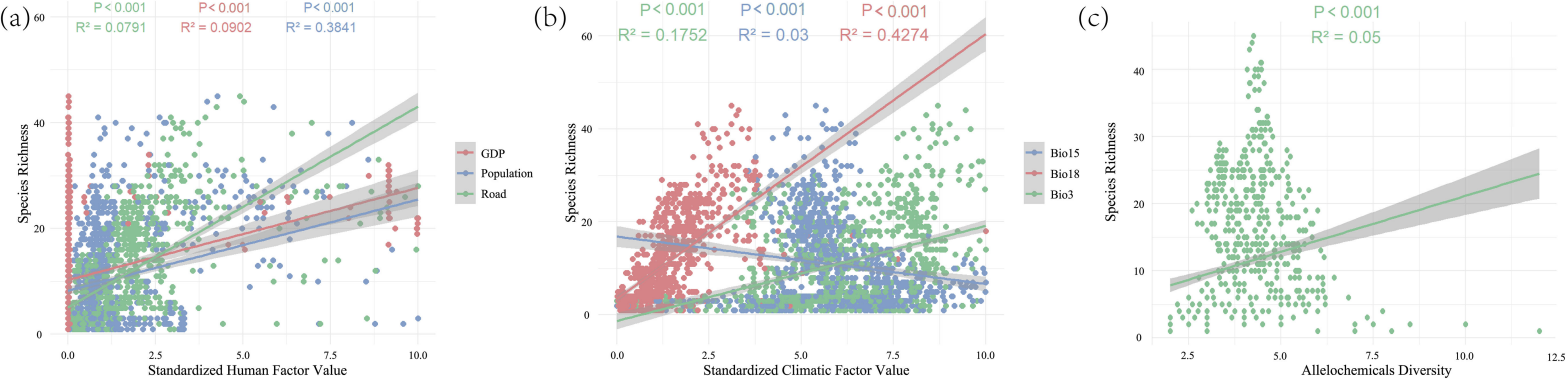
Ordinary least square regression results showing the effects of climatic, human, and allelochemical drivers on SR. **(a)** The human factor in SR regression; **(b)** The climatic factor in SR regression; **(c)** The allelochemicals in SR regression. R² represents the goodness of fit of a regression model to observed data, with values closer to 1 indicate a stronger model fit. Significance levels: *P < 0.05; **P < 0.01; ***P < 0.001. Human and climatic variables were standardized prior to analysis. Bio3: isothermality, Bio15: precipitation seasonality, and Bio18: precipitation of warmest quarter.

The seven driving factors influencing invasive plant species richness (SR) were categorized into three major groups: human activity variables (GDP, road density, and population), climatic variables (Bio3, Bio15, and Bio18), and allelochemical variables. Ordinary least squares (OLS) regression was applied to assess the impact on species richness (SR). The results showed that all three groups significantly affected SR (P < 0.001). Notably, Bio15 showed a negative correlation with SR, whereas all other factors displayed positive relationships (**Figure 3**). Overall, human, climatic, and allelochemicals exerted substantial effects on the spatial distribution patterns of invasive plants, corresponding to the spatial trends observed in the earlier analyses (**Figure 1**; **Supplementary Figures S1, S2**).

**Figure 4:**
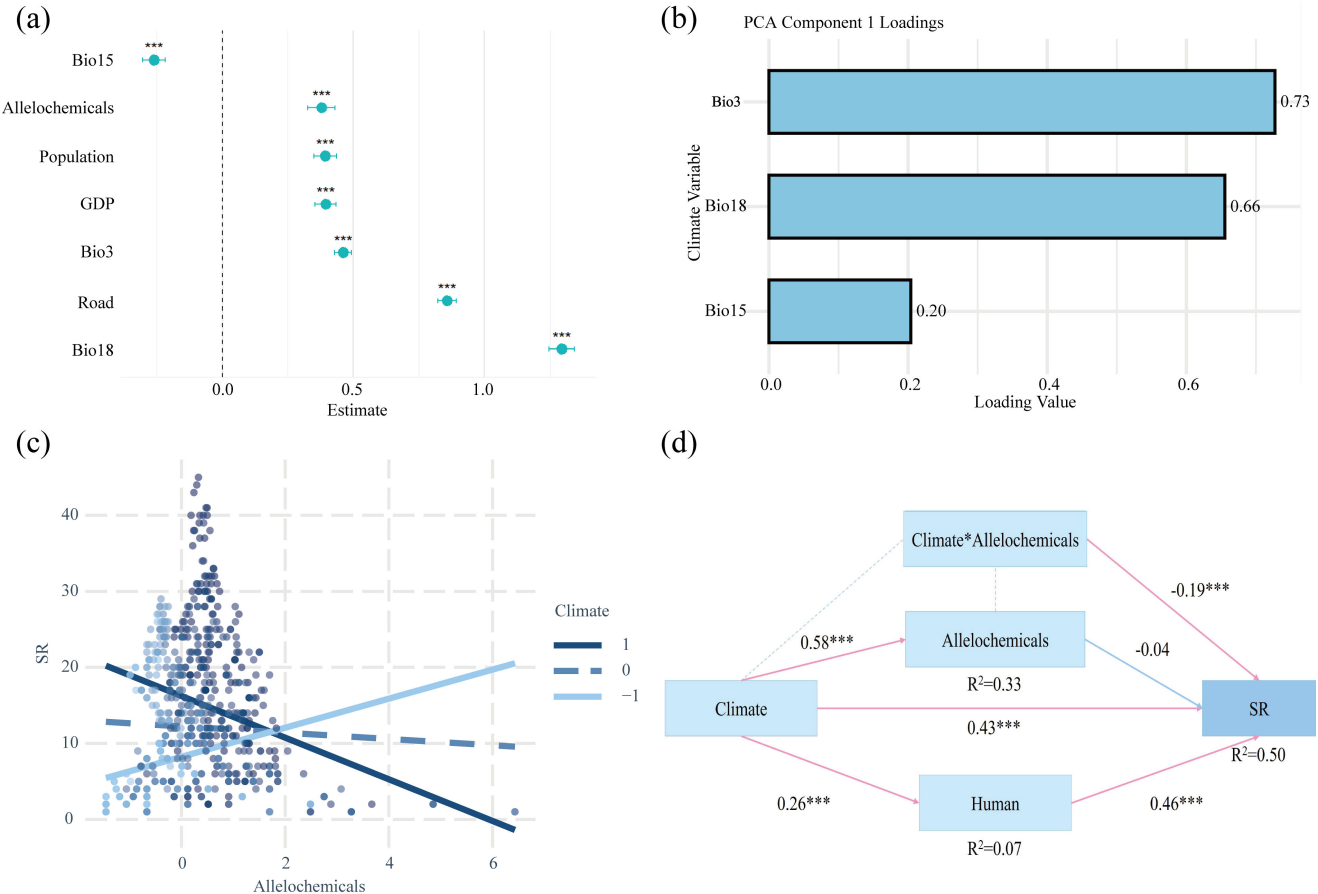
Results of mixed-effects model, interactions, and structural equation model (SEM). **(a)** Standardized effect sizes of different drivers on species richness. **(b)** Loading of climatic variables (Bio3, Bio15, and Bio18) on the first PCA axis. **(c)** Interaction between climate (the first PCA axis of bio3, bio15, and bio18) and allelochemical diversity on SR. **(d)** SEM of three key drivers on species richness. Significance levels: ***P < 0.001. Bio3: isothermality, Bio15: precipitation seasonality, Bio18: precipitation of warmest quarter.

The mixed-effects models revealed that Bio18, Bio3, road, GDP, population, and allelochemicals all exhibited significant positive effects on SR (P < 0.001), while Bio15 showed a significant negative effect (P < 0.001) (**Figure 4a**). A Bayesian model produced consistent results, further validating the robustness and reliability of our findings (**Supplementary Figure S3**). Our findings revealed a significant interaction between climatic conditions and allelochemical diversity. In stable humid environments characterized by high isothermality, high precipitation in the warmest quarter, and moderately high precipitation seasonality, allelochemical diversity was negatively correlated with native invasive plant diversity. In contrast, in arid inland regions with low isothermality, low warm-season precipitation, and moderately low precipitation seasonality, allelochemical diversity was positively associated with native invasive plant diversity (**Figures 4b, c**). Finally, our SEM revealed three major pathways (**Figure 4d**): 1) climatic factors were positively correlated with the intensity of human activity, 2) climate and human activity collectively determined native invasive plant diversity, and 3) the relationship between allelopathy and invasive plant diversity was indirectly mediated by climate.

### 3.3 Spatial structure of predictors

We calculated Moran’s I for each driving factor to better evaluate the influence of the selected variables. We compared the results of OLS regression with those from SAR. Moran’s I analysis revealed statistically significant spatial autocorrelation (P < 0.05) for all major drivers of invasive plant distribution on the QTP, such as allelochemicals, GDP, road density, population, Bio3, Bio15, and Bio18. All predictors had Moran’s I values greater than 0.79 (p < 0.05), aligning with their visually apparent spatial clustering patterns (**Figure 1a**). OLS regression results demonstrated that all factors were statistically significant, with positive relationships observed except for Bio15. However, when accounting for spatial dependence, the SAR model revealed that several predictors lost significance. This highlights the spatial clustering of both predictors and the response variable. The SAR model identified Bio15 as the dominant driver (pseudo R² = 0.248), followed by Population (pseudo R² = 0.242) and GDP (pseudo R² = 0.241), while Road and Bio18 exhibited moderate effects, and Allelochemicals showed the weakest influence (**Supplementary Tables S3, 4**). This spatial co-occurrence itself is an essential finding, reflecting the geographically structured interactions between native invasive plant species distribution and their environmental and human drivers.

### 3.4 Future expanding trends of native invasive plants

**Figure 5:**
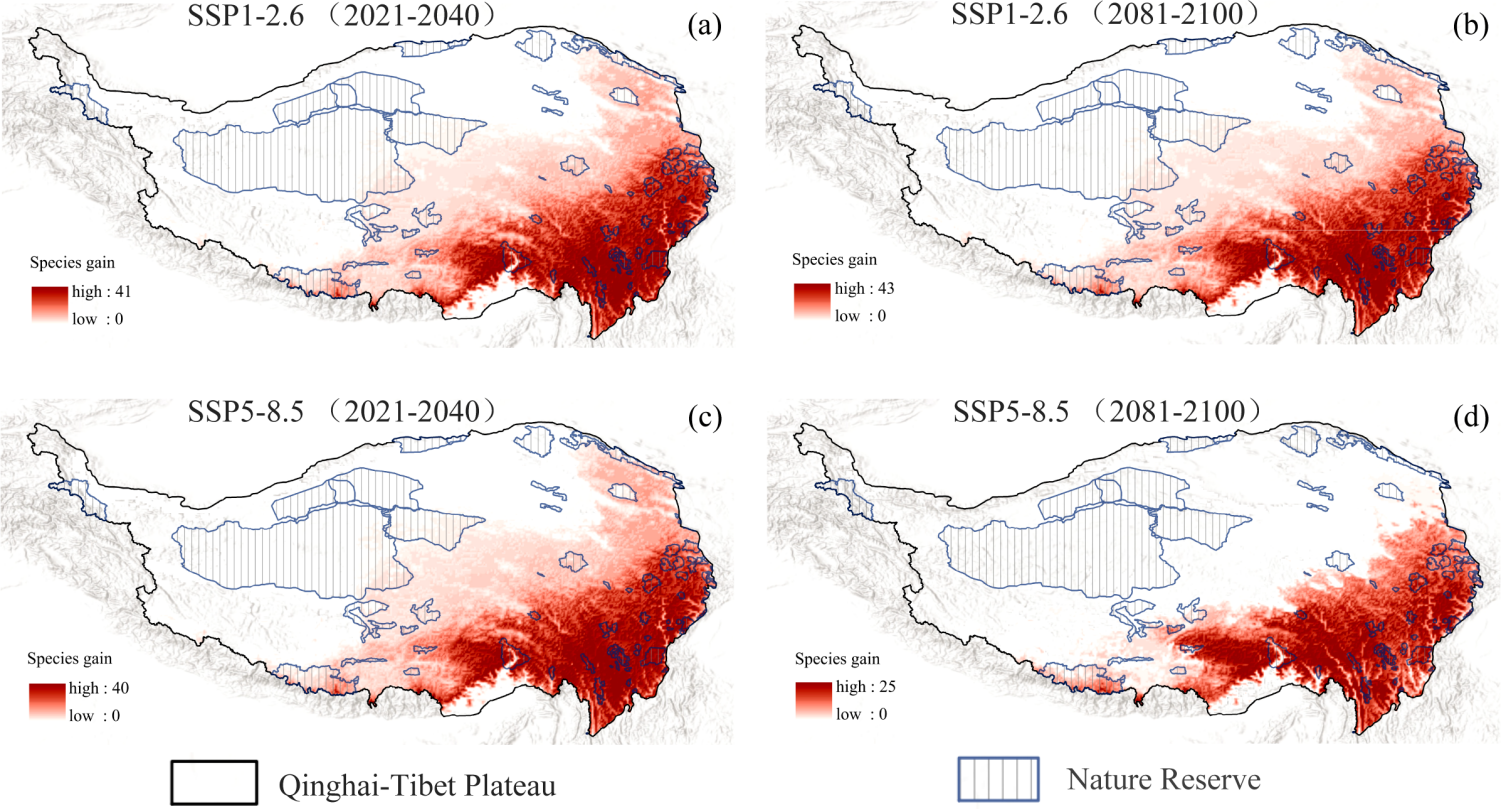
Projected expansion of native invasive plant species under future climate scenarios. **(a)** Projections for 2021–2040 under the SSP1-2.6 scenario. **(b)** Projections for 2081–2100 under the SSP1-2.6 scenario. **(c)** Projections for 2021–2040 under the SSP5-8.5 scenario. **(d)** Projections for 2081–2100 under the SSP5-8.5 scenario.

Under the SSP1-2.6 scenario, projections for both the near future (2021-2040) and the late century (2081-2100) indicate that the eastern, southeastern, and southern regions of the QTP will remain primary hotspots for native invasive plant expansion, with newly suitable habitat covering approximately 199.26 × 10⁴ km² (78.56% of the QTP) and 197.04 × 10⁴ km² (77.68%) (**Supplementary Table S5**), respectively. Notably, a significant portion of this expansion overlaps with protected areas (PAs), reaching 46.31 × 10⁴ km² (74.88% of total PA) in the near future and 44.56 × 10⁴ km² (72.04%) (**Supplementary Table S5**) by the end of the century, illustrating a consistent northwestward encroachment into PAs (**Figures 5a, b**). Under the high-emissions (SSP5-8.5) scenario, the expansion trend during 2021–2040 remained comparable, with 197.62 × 10⁴ km² (77.91%) of newly suitable habitat, including 46.00 × 10⁴ km² (74.38%) (**Supplementary Table S5**) overlapping with PAs. However, by 2081-2100, the total expansion area decreased substantially to 121.29 × 10⁴ km² (47.82%), and overlap with PAs dropped markedly to 14.58 × 10⁴ km² (23.57%) (**Supplementary Table S5**), although existing hotspots in the eastern, southeastern, and southern hotspots persisted (**Figures 5c, d**). These results quantitatively highlight that under sustained low-emission scenarios, invasive hotspots are not only likely to intensify but also increasingly encroach into northwestern protected zones, whereas high-emission scenarios may restrict overall expansion but still sustain significant pressure in strategic regions.

## 4 Discussion

### 4.1 Synthesis of key findings: current patterns and future dynamics

Our study reveals that native invasive plants on the QTP exhibit a spatially clustered distribution, concentrated primarily in the eastern and southeastern regions. This pattern aligns with established biogeographical gradients of overall plant diversity on the plateau (Zhang et al., 2016; Yu et al., 2018) and reflects the synergistic operation of climatic, anthropogenic, and allelochemical drivers. The convergence of high native invasive plant diversity with general biodiversity hotspots and human population centers (Luck, 2007) creates compounded conservation challenges that extend beyond simple species co-occurrence to fundamentally alter ecosystem vulnerability. Notably, current invasion hotspots overlap strongly with areas projected to be at high risk under future climate scenarios, suggesting escalating conservation challenges. Our projections indicate not only the persistence of current southeastern hotspots but also significant northward and westward expansion of invasion risks, including encroachment into several protected areas. This spatial-temporal dynamic underscores the urgent need for proactive management strategies that address the interacting effects of multiple drivers in this ecologically sensitive region.

### 4.2 Mechanistic interpretation of interactive drivers

Our findings extend invasion ecology theory by demonstrating how multiple drivers operate interactively rather than independently. While previous research has often treated climate, human activities, and allelopathy as separate factors (Inderjit et al., 2011), our integrated analysis reveals they form a coherent causal chain: climatic stability sets the fundamental niche space, human disturbance creates invasion opportunities and dispersal pathways, and allelochemical diversity determines competitive outcomes. This multi-driver perspective helps resolve apparent contradictions in native invasion patterns and provides a more predictive framework for understanding native invasion dynamics in complex mountain ecosystems.

#### 4.2.1 Climate as a selective filter and enabler

The observed climatic drivers reveal how environmental stability and resource availability shape invasion patterns across the QTP. The positive correlations with Bio3 (isothermality) and Bio18 (precipitation of warmest quarter) demonstrate that climatic stability functions as a selective filter that differentially favors native invasive plants. High isothermality buffers thermal fluctuations, enhancing environmental tolerance and promoting niche differentiation in topographically complex regions like the Hengduan Mountains (Zhang et al., 2021a), while reliable warm-season precipitation supports establishment during critical growth periods. Conversely, the negative correlation with Bio15 (precipitation seasonality) reflects the ecological constraints of temporal resource variability, where high intra-annual precipitation fluctuations increase environmental stress and limit invasion success (Bradford and Lauenroth, 2006; Zhong et al., 2025).

Critically, climate change amplifies these dynamics by simultaneously expanding suitable habitats for native invasive plants while stressing native communities. Our projections indicate southeastern QTP will remain highly vulnerable with significant increases in invasion intensity, while new hotspots emerge northward and westward, including encroachment into protected areas. This spatial restructuring underscores the urgency of integrating climate forecasts into conservation planning, enabling proactive management that anticipates range shifts rather than merely responding to established native invasions. The demonstrated association between climate suitability and native invasion risk validates the utility of climate-based forecasting while highlighting the need for adaptive strategies that address the dynamic nature of invasion processes under rapid environmental change.

#### 4.2.2 Human activity as a distribution catalyst

Human disturbances significantly alter invasion trajectories through multiple synergistic pathways. Large-scale infrastructure development degrades ecosystem integrity through vegetation clearance, soil erosion, and habitat fragmentation (Yang, 2015), while transportation networks create continuous dispersal corridors for invasive propagules (Hulme, 2009). The operational phase of infrastructure projects particularly accelerates species spread by enabling rapid colonization of opportunistic species like *Galinsoga parviflora* and *Bidens pilosa* in disturbed environments such as dry-hot valleys (Chen et al., 2025). This human-mediated dispersal interacts strongly with pre-existing biodiversity patterns, establishing invasion feedback loops where disturbed high-biodiversity areas experience disproportionately severe invasion impacts (Colautti et al., 2006).

Beyond local ecosystem degradation, these invasions pose substantial threats to regional biodiversity, ecosystem services, and socioeconomic stability (Deng et al., 2020). Mounting empirical evidence confirms that human-mediated disturbances represent one of the most potent drivers of biological invasions worldwide (van Kleunen et al., 2018), with this study demonstrating their equal relevance for native invasive plants. The spatial concentration of human activities in southeastern QTP, consistent with documented demographic shifts (Zheng, 2024), creates particularly pronounced native invasion risks. This pattern is exemplified by species like *Galinsoga quadriradiata*, whose occurrence frequency peaks in areas of intensive human activity (Chen et al., 2025), demonstrating clear links between disturbance intensity and invasion pressure.

These findings underscore the critical need to regulate both the intensity and spatial extent of human activities, particularly in ecologically sensitive regions. Future ecosystem management must prioritize areas of concentrated human activity for targeted monitoring and intervention, developing integrated approaches that address the multiple mechanisms through which human disturbance facilitates invasion across different stages from initial dispersal to landscape-scale establishment.

#### 4.2.3 Allelochemical diversity as a novel weapons portfolio

Our findings demonstrate that allelochemical diversity significantly shapes native invasion patterns across the QTP by creating a heterogeneous “chemical landscape” that operates at regional scales. The Novel Weapons Hypothesis (Hierro and Callaway, 2003) provides a mechanistic basis for understanding how invasive plants employ diverse chemical compounds unfamiliar to local communities, disrupting plant-soil feedbacks and microbial interactions (Karban, 2007; van Kleunen et al., 2018). Species such as *Euphorbia jolkinii* exemplify this mechanism, where potent allelochemicals degrade alpine meadows by altering community structure and reducing biodiversity (Dai et al., 2022).

While most previous studies have focused on small-scale or single-species allelopathic effects (Inderjit et al., 2011), our research reveals that regional allelochemical diversity, representing the collective chemical repertoire across landscapes, enhances the species pool of native invasive plants. This broader perspective offers valuable insights into ecosystem-level management. Notably, allelochemical effects are context-dependent, interacting strongly with climatic conditions. In arid inland regions, reduced microbial degradation and heightened plant stress may amplify the impact of chemical interference, highlighting the need to investigate climate-chemistry-invasion interactions more deeply.

### 4.3 Limitations and causal uncertainties

Several limitations and uncertainties warrant careful consideration when interpreting our results and projections. First, while we applied stringent criteria for identifying native invasive species, limited regional research and incomplete distribution data may have resulted in underestimation of certain invasion risks. Second, our future projections carry inherent uncertainty as they are based primarily on climate scenarios; this approach excludes future changes in human activities and allelochemical dynamics, which may lead to an underestimation of invasion potential, particularly in regions undergoing rapid socioeconomic development or chemical landscape alteration. Third, the relationship between allelochemical diversity and invasion patterns, while statistically significant, represents correlation rather than confirmed causation; the direction of influence between chemical diversity and invasion success requires experimental verification. Finally, our macroecological approach necessarily simplifies complex species interactions and local-scale processes that modulate invasion outcomes. Future research should integrate metabolomic profiling with manipulative experiments to establish causal pathways and quantify interaction strengths among drivers, while incorporating scenarios of human disturbance and chemical evolution to improve the robustness of invasion forecasts.

### 4.4 Management implications and future directions

The spatial explicitness of our findings enables targeted management strategies. First, southeastern hotspots require immediate containment efforts focused on transportation corridors and areas of high human activity. Second, projected expansion zones in northern and western regions need proactive monitoring, particularly within protected areas vulnerable to future invasion. Third, allelochemical diversity mapping can refine risk assessments and guide the development of biocontrol approaches using allelopathy-resistant forage grasses (Jabran and Farooq, 2013) and compound-degrading microbes (Liu et al., 2023, 2024). Future research should prioritize quantifying interaction effects among drivers, particularly how climate change modulates allelochemical efficacy and how human disturbance intensity alters invasion thresholds. By addressing these priorities, we can develop more resilient conservation strategies for the QTP’s unique ecosystems in an era of rapid environmental change.

The ecological patterns and management implications identified on the Qinghai–Tibet Plateau find parallels in other high-elevation and mountainous systems worldwide, underscoring the broader relevance of our findings. In the Mongolian Plateau, *Stellera chamaejasme* has demonstrated significant invasive potential through pollen allelopathy, toxicity to livestock, and intense resource competition, leading to extensive grassland degradation (Sun et al., 2010; Guo and Wang, 2018). Similarly, in Brazil, native woody bamboos have proliferated rapidly in fragmented habitats (Pivello et al., 2018), while in western U.S. grasslands, *Juniperus occidentalis* has expanded its range through interactions with grazing and altered fire regimes (Miller et al., 2005). These cases collectively highlight that the interplay of climate change, human disturbance, and allelochemicals as emphasized in our study, also drives native invasive plants across diverse mountainous regions. Consequently, the integrative management framework we propose, which emphasizes spatial prioritization, allelochemical monitoring, and adaptive intervention, holds promise for application in other highland ecosystems facing similar invasion threats.

## 5 Conclusions

Our study provides the first large-scale evidence that native invasive plants on the QTP exhibit a spatially clustered distribution, with concentrations primarily in the eastern and southeastern regions. These invasion hotspots align with zones of high biodiversity and intense human activity and underscore the synergistic effects of climatic complexity, human disturbance, and regional allelochemical diversity in driving invasion risks.

Our findings provide several key innovations to the field of invasion ecology: (1) Revealing the role of allelochemical diversity in large-scale invasion patterns. We demonstrate that regional diversity of allelochemicals significantly enhances the species pool of native invasive plants, a mechanism previously underexplored in macroecological invasion studies. (2) Integrating multiple drivers of native invasive plant patterns. By combining climatic variables, human disturbance, and allelochemical diversity, our study moves beyond traditional frameworks that focus primarily on climate, human factors, and exotic species, offering a more comprehensive understanding of native invasive plant distributions. (3) Proposing novel, mechanism-based management strategies. Our findings have important implications for managing ecosystems under global change. Specifically, we suggest (1) prioritizing highly disturbed areas (e.g., the Hengduan Mountains, the Linzhi area in southeastern Xizang, and the Qiangtang Plateau) for early detection and rapid intervention of high human activities, (2) incorporating climatic stability and precipitation variability into predictive models to better predict future invasion risks, and (3) developing region-specific biocontrol measures that target allelopathic suppression mechanisms, thereby safeguarding biodiversity and enhancing ecological resilience. We recommend tools such as allelopathic fingerprinting to identify invasion hotspots and biocontrol approaches that use allelochemical-degrading microbes or resistant forage grasses to disrupt the synergistic expansion of native invasive plants.

By bridging large-scale spatial analyses with underlying biochemical and ecological mechanisms, this study reshapes our understanding of the patterns and drivers of native invasive plants. It also offers a holistic, scalable framework for invasive species management and ecological restoration on the QTP and in other climate-sensitive and biodiversity-rich regions.

## Data Availability

The original contributions presented in the study are included in the article/**Supplementary Material**. Further inquiries can be directed to the corresponding authors.
