# Supplementary material for: Diversity patterns, abiotic and biotic drivers, and future dynamics of native invasive plants on the Qinghai-Tibet Plateau
Source: Front Plant Sci. 2025 Nov 13;16:1715360. doi: 10.3389/fpls.2025.1715360 (PMC12659197; doi:10.3389/fpls.2025.1715360)

**Supplementary information**

**Table S1. Checklist of native invasive plants on the Qinghai-Tibet Plateau.**

*Note: The current checklist was primarily compiled based on field surveys, literature review, and allelopathic effects. However, the invasive status of a small number of species requires further investigation and more definitive data for accurate classification in the future.*

| **Species** | **Allelochemical** | **Location** | **References** |
| --- | --- | --- | --- |
| *Morina kokonorica* | Lipids and lipid-like molecules | Gansu, Qinghai, Sichuan, Xizang | Lv, C. P. (2008). Study on the Chemical Constituents of *Morina kokonorica*. Lanzhou University. |
| *Phlomoides rotata* | Flavonoids, Terpenoids, Shikimates and phenylpropanoids | Shangri-La, Gansu | XIE, T. P., CUI, Z. J., HUANG, Y. F., LI, Y., & ZHANG, X. H. (2023). Allelopathic Effects of *Lamiophlomis rotata* on Seed and Seedlings of Itself and Two Crops in Alpine Areas. Chinese Journal of Grassland, 45(07):30-40. Li, J. W., Bi, Y. Y., & Li, J. J.​(2024). Research Progress on Chemical Constituents and Pharmacological Effects of Tibetan Medicine *Lamiophlomis rotate* and Its Quality Marker Prediction Analysis. Modern Chinese Medicine, 26(5):903-911. |
| *Clinopodium repens* | Flavonoids, Terpenoids, Phenols | Gansu, Sichuan, Yunnan | Dai, J. R., Shi, D. W., & Zhang, H. C.​​ (1984). MORPHOLOGY, ANATOMY AND CHEMICAL CONSTITUENTS OF FIVE SPECIES OF CLINOPODIUM. Acta Pharmaceutica Sinica, (06):425-430+482-484. |
| *Ajuga lupulina* | Organic acids | Gansu, Sichuan, Xizang, Qinghai | Tang, Y.​ (2008). A Allelopathic effects of *Aerba lupulin* and *Aconitum pendulum* of ‘black soil patch’ degraded Alpinemeadow on herding grasses. Qinghai University.  ​​Tang, Y., Shang, Z. H., Li, X. L., Ma, Y. S., Wu, H. Y., & Long, R. J. (2008). Allelopathic effects of *Aerba lupulin* on several graminaceous grass species on alpine meadow. Chinese Journal of Ecology, 27(12):2067-2072. |
| *Nepeta hemsleyana* | Shikimates and phenylpropanoids, Terpenoids, Flavonoids, Lipids and lipid-like molecules | Gyantse and Lhasa, Xizang | Meng, N., Huang, S., Hu, D. D., Xu, Y. L., Wang, Y. F., & Wang, J. L. (2017). Chemical constituents from Nepeta angustifolia. Chinese Traditional Patent Medicine, 39(05):976-980. |
| *Chelonopsis souliei* | Lipids and lipid-like molecules, Shikimates and phenylpropanoids | Western Sichuan, Xizang | ​Chen, X. H., & Peng, C. (2009). Studies on the Chemical Constituents of *Chelonopsis albiflora*. Journal of Chinese Medicinal Materials, 32(03):365-367. |
| *Leucosceptrum canum* | Terpenoids, Flavonoids, Shikimates and phenylpropanoids, Carbohydrates, Phenols, Lipids and lipid-like molecules | Yunnan, Southwestern Sichuan | Xiong, R. Q., Jiang, J. H., Luo, Y. H., & Mu, Q. S. (2013). Advance on the Chemical and Bioactive Studies of *Leucosceptrum*. Journal of Anhui Agricultural Sciences, Journal of Anhui Agricultural Sciences, 41(08):3374-3376. |
| *Origanum vulgare* | Shikimates and phenylpropanoids, Terpenoids, Phenols | Sichuan, Yunnan, Gansu, Xizang | Zhao, Y., Yang, Y. H., Wang, K. B., Fan, L. M., Su, F. W., & Ye, M. (2020). Chemical composition and allelopathic potential of essential oil isolated from *Origanum vulgare*. Chinese Journal of Applied Ecology, 31(07): 2257-2263. |
| *Isodon wardii* | Alkaloids | Southeastern Xizang | Fan, Z. T., Chen, J. R., Jiang, S. P., Zhang, H. J., & Zhao, Q. S. (1996). Analysis of Diterpenoids in *Isodon amethystoides* from Sichuan-Xizang. Xizang Science and Technology, 46(04), 37–38. |
| *Elsholtzia densa* | Flavonoids | Batang County, Garzê Xizangan Autonomous Prefecture, Sichuan; Qinghai | Chen, Y., Zhang, Y. X., Guo, L. Z., & Wei, Y. H. (2022). Autotoxic effects of water extract of *Elsholtzia densa* Benth. on seed germination and seedling growth. Journal of Qinghai University, 40(02): 48-54.  Chen, Y. (2022). Study on interspecific relationship between *Chenopodium album* and *Elsholtzia densa* in *Chinese Brassia* *campestris* field. Qinghai University, |
| *Galeopsis bifida* | Flavonoids, Carbohydrates | Qinghai, Gansu, Western Sichuan, Yunnan, Xizang | Li, W. D., & Zhang, Y. H. (2003). Flavonoid glycosides from *Galeopsis bifida*. Acta Botanica Boreali-Occidentalia Sinica, 23(04), 637–640. |
| *Euphorbia jolkini* | Phenols | Shangri-La | Wang, Z. Y., Liu, X. Y., Zhou, S. L., & Li, W. (2022). Allelopathic Effects of Dominant species *Euphorbia jolkinii* on Three Receptor Plants in Alpine Degraded Meadow. Acta Agriculturae Universitatis Jiangxiensis, 44(03), 635–646.  ​​Niu, Q. M., Yang, X., Ma, Z. Y., Chu, X. H., Luo, Q., Liu, Y., Xie, Y., Liu, J., & Shan, G. L. (2024). Study on the Allelopathic Effects of Extracs from the Roots of *Euphorbia jolkinii* on Three High Quality Forages. Chinese Journal of Grassland, 46(01), 135–142. |
| *Tibetia yunnanensis* | Alkaloids, Flavonoids | Shangri-La, Western Sichuan, Xizang | Xie, Y. P., Sun, H., & Nie, Z. L. (2012). Pollen Morphology of *Tibetia* ( Fabaceae) from the Hengduan Mountains，with Emphasis on the Taxonomical Status of *Tibetia liangshanensis*. Plant Diversity and Resources, 34(4), 326–332. |
| *Sophora alopecuroides* | Alkaloids | Gansu, Qinghai, Xizang | Shi, G. Q., Sui, X. Q., Yang, J., Liu, Y., Cui, G. Y., & Zhang, B. (2022). Allelopathic Effects of Extracts from Roots, Stem and Leaves of *Sophora alopecuroides* on Seed Germination of Four Forage Species. Acta Agrestia Sinica, 30(08), 2223–2230. |
| *Astragalus yunnanensis* | Flavonoids | Shangri-La, Yunnan | Gong, G. F., Dou, Z. R., Zhou, G. J., et al.​​ (2023). Species diversity and anti-biofilm components of rhizosphere microorganisms derived from *Astragalus forrestii* in special high-cold environments. Acta Microbiologica Sinica, 63(10), 3967–3986. |
| *Oxytropis ochrocephala* | Terpenoids, Alkaloids | Gannan Xizangan Autonomous Prefecture, Gansu | Hou, Y., Cheng, X. Y., Ren, G. H., Deng, B., & Shang, Z. H. (2011). Allelopathic Effects of the Typical “Black Soil Land” PoisonousPlant on *Pedicularis kansuensis* in Qinghai-Tibetan Plateau. Acta Botanica Boreali-Occidentalia Sinica, 31(8), 1651–1656.  ​​Fan, P. H. (2014). Research for Seed Dormancy Breaking and Allelopathic of *Oxytropis ochrocephala* Bunge [D]. Northwest University, 36(9), 74. |
| *Oxytropis glabra* | Terpenoids | Gansu, Qinghai, Xizang | Xiao, H., Wang, F., Xu, C. L., Zhang, J. W., Yang, H. L., Chai, J. L., & Yu, X. J. (2017). Effects of Rhizosphere Soil of Achnatherum inebrians on Seed Germination and Seedling Growth of Six Plant Species in Alpine Meadow. Grassland and Turf, 37(1), 38–43.  ​​Xia, C., Zhong, R., Zhang, X. X., & Nan, Z. B. (2015). Achnatherum Effects of Volatile Compounds from Endophyte-Free and infected *Achnatherum Inebrians* on *Lolium Perenne*. Pratacultural Science, 32(5), 658–666. |
| *Thermopsis lanceolata* | Flavonoids, Carbohydrates, Phenols, Organic acids, Alkaloids, Lipids and lipid-like molecules, Terpenoids | Gansu, Xizang, Sichuan | Li, Y., Zhao, X. R., & Zhang, X. L. (2008). Study on chemical components of *Thermopsis lanceolata*. Journal of Agricultural Science Research, 29(4), 68–69. |
| *Elymus nutans* | Flavonoids | Gansu, Qinghai, Sichuan, Xizang | ​Liang, J. (2020). Allelopathic Potential of Major Poisonous Weeds and Grasses in Alpine Meadows. Qinghai University. |
| *Festuca coelestis* | Terpenoids, Phenols | Qinghai | ​Zhang, H., Ye, Y. H., Li, W. C., et al.​​ (2024). Effects of water extracts from litter of alpine meadows and grasslands innorthern Tibet on seed germination. Pratacultural Science, 1-13. [Accessed 2024-08-10]. http://kns.cnki.net/kcms/detail/62.1069.S.20240618.1819.002.html. |
| *Hippophae rhamnoides subsp. yunnanensis* | Alkaloids | Northwestern Yunnan, South of Kangding, East of Lhasa | Yang, H., Gao, W. Y., Lv, Q. X., Bai, Y. Z., Ruan, C. J., Ding, J., & Sun, Z. J. (2024). Ecological Stoichiometry of Carbon, Nitrogen, and Phosphorus in *Hippophae rhamnoides* Leaves and Soil Across Different Habitats on the Qinghai-Tibet Plateau [J]. Journal of Northeast Forestry University, 52(8), 110–119. |
| *Hippophae gyantsensis* | Alkaloids | Lhasa, Gyantse, Yadong, Xizang | Yang, H., Gao, W. Y., Lv, Q. X., Bai, Y. Z., Ruan, C. J., Ding, J., & Sun, Z. J. (2024). Ecological Stoichiometry of Carbon, Nitrogen, and Phosphorus in *Hippophae rhamnoides* Leaves and Soil Across Different Habitats on the Qinghai-Tibet Plateau [J]. Journal of Northeast Forestry University, 52(8), 110–120. |
| *Hippophae tibetana* | Alkaloids | Gansu, Qinghai, Sichuan, Xizang | Yang, H., Gao, W. Y., Lv, Q. X., Bai, Y. Z., Ruan, C. J., Ding, J., & Sun, Z. J. (2024). Ecological Stoichiometry of Carbon, Nitrogen, and Phosphorus in *Hippophae rhamnoides* Leaves and Soil Across Different Habitats on the Qinghai-Tibet Plateau [J]. Journal of Northeast Forestry University, 52(8), 110–121. |
| *Astilbe rivularis* | Terpenoids, Phenols | Northwestern Yunnan, Xizang, Sichuan | Wei, Q. H., Ruan, Z. G., Liao, X. R., Tang, Z. M., & Dai, Q. F. (2003). Study on Pharmacognosy of *Astilbe rivularis* Buch.-Hame.ex D Don. Journal of Yunnan University of Chinese Medicine, 26(2), 21–23+40. |
| *Peganum harmala* | Alkaloids | Hexi Corridor, Gonggar, Zedang | Wang, F. L., Liu, S. Z., Wei, Q. S., Wei, X. H., Chai, C. W., Li, A. D., Zhang, Y. H., Wang, Y. Q., Wang, F., & Zhang, J. C. (2017). Allelopathy of Setm-leaf Aqueous Extracts of *Peganum harmala* to Seed Germination and Seeding Growth of Lycium ruthenicum. Journal of Desert Research, 37(03), 469–474. |
| *Peganum multisectum* | Alkaloids, Phenols | Qinghai, Gansu | Liu, J. X., Hu, H. B., & Wang, X. (2008). Study on Allelopathy of Aqueous Extract from *Peganum multisectum*（Maxim．） Bobr．on Perennial Ryegrass （*Lolium perenne* L．）and Its Physiological-biochemical Manifestation. Acta Agrestia Sinica, 16(4), 374–379. Zhang, F. Q., Fu, P. C., Gao, Q. B., Li, Y. H., Gulzar Khan, & Chen, S. L. (2013). Comparative Study on Plant Seed Morphological Characteristics of Zygophyllaceae and Two New Families Separated from It. Plant Diversity and Resources, 35(03), 280–284. |
| *Erigeron multiradiatus* | Flavonoids, Lipids and lipid-like molecules, Phenols | Garzê, Northwestern Yunnan, Xizang | Wang, J. G., Zhang, Z. F., Li, J., Jiang, H., Peng, H. C., & Zhang, H. (2008). Chemical constituents from *Erigeron multiradiatus* extracted with ethylacetate. West China Journal of Pharmaceutical Sciences, 23(3), 255–257. |
| *Saussurea pulchra* | Flavonoids, Phenols | Qinghai, Deqin, Xizang | Zhu, P. J., Yang, L., Shi, S. B., & Han, F.​​ (2013). Comparison of Physiological and Biochemical Characteristics of *Saussureapulchra* from Different Altitudes in Tibet Plateau. Journal of Green Science and Technology, (07), 54–57. |
| *Artemisia nanschanica* | Lipids and lipid-like molecules, Others | Xizang | Nima Zhuoga. (2019). Decomposition of *Artemisia nanshanensis* Residues in Soil [J]. Xizang Science and Technology, 41(2), 5–9. Chen, Y. C., Ma, S. Q., & Lu, X. Y. (2019). Litter decomposition and nutrient dynamics of typical plant species in an alpine steppe ecosystem in Northern Tibet. Pratacultural Science, 41(19), 1066–1073. |
| *Leontopodium nanum* | Flavonoids | Xizang, Western/Northwestern Sichuan, Qinghai | Liang, J. (2020). Allelopathic Potential of Major Poisonous Weeds and Grasses in Alpine Meadows. Qinghai University. |
| *Ligularia cymbulifera* | Shikimates and phenylpropanoids, Phenols | Shangri-La | Wang, H., Ma, X. L., Ling, R. G., Zhang, Y., Ren, J., & Dai, W. R. (2022). Allelopathic Effects of *Ligularia Cymbulifera* Extracts on Seed Germination and Seedling Growth of Three Forages. Acta Agrestia Sinica 2022, Vol. 30 Issue (1): 93-99. |
| *Ligularia duciformis* | Shikimates and phenylpropanoids, Lipids and lipid-like molecules | Garzê, Shangri-La | Gao, K., & Jia, Z. J. (2000). Structural Study of a New Compound from *Ligularia duciformis* Journal of Lanzhou University. 2000,(01):131-132. |
| *Ligularia sagitta* | Terpenoids, Alkaloids | Qinghai, Shangri-La | Ma, R. J., Li, G., Zhu, H., Zhang, H., & Wang, N. L. (2007). Allelopathic effects of aqueous extracts from *Ligularia sagitta* on seed of nine pasture plants . Acta Prataculturae Sinica, ,2007,(06):88-93.  Li, X. W., Sun, K., Ma, R. J., & Cao, Y. (2024). Gannan natural grassland poisonous plants and prevention and cure countermeasures. Pratacultural Science. 2003,(10):60-63. |
| *Ligularia virgaurea* | Flavonoids, Terpenoids, Alkaloids | Shangri-La, Huangnan Xizangan Autonomous Prefecture | Ma, R. J., et al. (2005). Allelopathy and chemical constituents of *Ligularia virgaurea* volatile. Chinese Journal of Applied Ecology 2005,(10):1826-1829.  Tang, Y. L., Deng, Y. R., & Wang, H. Q. (2003). Chemical Constituents of Essential Oil from *Ligularia virgaurea*. China Journal of Chinese Materia Medica, 28(7), 627–629. |
| *Ligularia dictyoneura* | Flavonoids, Terpenoids | Shangri-La, Sichuan, Kunming | Niu, Q. M., Shan, G. L., Luo, Q., Liu, Y., Liu, J., Xie, Y., Deng, S. C., Yang, X., Chu, X. H. (2023). Effect of Invasion and Diffusion of Poisonous Weeds on Soil Microbial Diversity in Subalpine Meadow in Northwest Yunnan. Acta Agrestia Sinica, 2023, Vol. 31 Issue (7): 1996-2004.  Tan, A. M., Wang, Z. T., Hong, X., Li, S. L., & Hao, X. J. (2002). Study on the Chemical Constituents of *Ligularia dictyoneura*. Journal of China Pharmaceutical University, 24(15), 30–40. 2002, (02):26-28. |
| *Ligularia vellerea* | Phenols | Lijiang, Zhongdian, Deqin | XI Zeng pu, DU Fan, WANG Jian. A Phytosociological Study on Subalpine Degraded Meadow in Shangri La County. Journal of Southwest Forestry University, 2013, 33(2): 75-80.  Li, Y. S., Luo, S. D., Zhang, M., Chen, J. J., & Wang, Z. T. (2001). Chemical Constituents of Ligularia gossypina. China Journal of Chinese Materia Medica, (12). |
| *Ligularia hodgsonii* | Terpenoids, Alkaloids, Carbohydrates, Lipids and lipid-like molecules, Flavonoids | Yunnan, Southwestern Gansu, Sichuan | Wang, J. B., Ge, Q., Huang, Y. Y., & Wang, Z. Z. (2013). Optimization of Extraction Process for Total Flavonoids from *Ligularia hodgsonii*. Chinese Journal of Spectroscopy Laboratory 2013,30(04):1792-1795. |
| *Ligularia tongolensis* | Flavonoids | Shangri-La, Xizang, Sichuan | Wang, J. F., Kuroda, C., & Gong, X. (2007). Assessment of Genetic Variation and Differentiation of Ligularia tongolensis (Compositae) Detected by ISSRs. Plant Diversity. 2007,(05):537-542. |
| *Ligularia paradoxa* | Flavonoids, Alkaloids | Northwestern Yunnan, Xizang, Qinghai, Sichuan | Zhang, R., Feng, X. Y., & Gong, X.(2015). The Transferability of SSR Markers in Seven Asteraceae Species to Taxa in the Natural Hybrid Zone between *Ligularia duciformis* and *L.paradoxa*. Plant Diversity. 2015,37(04):416-422. |
| *Ligularia lamarum* | Shikimates and phenylpropanoids, Terpenoids | Sichuan, Xizang, Northwestern Yunnan | Yoshinori Saito，Motoki Hidaka，Akané Fukuda，Yasuko Okamoto，Katsuyuki Nakashima，Motoo Tori，Ryo Hanai，Xun Gong，Eiji Watanabe，Chiaki Kuroda. (2017) Intra-specific diversity of the chemical composition of Ligularia lamarum in the Hengduan Mountains, China: The structures of four new eremophilanes and a new seco-eremophilane. Phytochemistry Letters, 20:139-145 |
| *Ligularia × maoniushanensis* | Flavonoids, Alkaloids | Northwestern Yunnan, Xizang, Qinghai, Sichuan | Zhang, R., Feng, X. Y., & Gong, X.(2015). The Transferability of SSR Markers in Seven Asteraceae Species to Taxa in the Natural Hybrid Zone between *Ligularia duciformis* and *L.paradoxa*. Plant Diversity. 2015,37(04):416-422. |
| *Ligularia caloxantha* | Alkaloids, Flavonoids, Terpenoids | Deqin | Wu, D. Y. (2006). The Chemieal Constituents of *Ligularia caloxantha* (Diels) Hand一Mazz. and Studies on Tissue Cultural of *Ligularia jaPoniea* (Thunb.) Less Var *scaberrxm* a (Hayuta) Ling. Zhejiang University.2006 |
| *Ligularia nelumbifolia* | Alkaloids, Flavonoids | Shangri-La | Hu, L. (2021). Gene Introgression and Genetic Structure of Sympatric *Ligularia* and *Cremanthodium.* University of Chinese Academy of Sciences. |
| *Ligularia lidjiangensis* | Flavonoids, Terpenoids | Northwestern Yunnan, Lijiang | Ma, Y., Dai, W. F., Yuan, C., et al. (2024). Sesquiterpenoid chemical constituents of methanol extract from the whole plant of *Ligularia lidjiangensis*. Journal of Plant Resources and Environment, 2024,33(03):107-112. |
| *Ligularia veitchiana* | Terpenoids | Northwestern Yunnan | Zhao, Q. Q., Wang, J., Wang, X. Y., et al. (2019). Study on chemical constituents of *Ligularia veitchiana*. Chinese Traditional and Herbal Drugs, 2019,50(21):5212-5216.  Liu, Q. (2010). Studeis on the Structures and Bioactivities of Secondary Metabolites from Three Southern Plants. Lanzhou University. 2010 |
| *Ligularia kanaitzensis* | Alkaloids, Phenols | Northwestern Yunnan | Li, Y. S., Wang, Z. T., Zhang, M., et al. (2002). Study on identification of terpenes from *Ligulria kanaitzensis*. Chinese Pharmaceutical Journal, 2002 (01), 14–16. |
| *Ligularia lapathifolia* | Terpenoids, Alkaloids, Phenols, Flavonoids | Northwestern Yunnan | Fei, D. Q. (2008). Studeis on the Chemical Constituents and Their Bioactivities of Three Compositae and One Leguminosae Species. Lanzhou University. 2008 |
| *Ligularia stenocephala* | Flavonoids, Terpenoids | Xizang | Lu, T., & Lin, X. Z. (2007). Seed Germination Characteristics of Two Ligularia eSpecies [J]. Journal of Zhejiang Forestry Science and Technology, (06), 37–40. |
| *Ligularia discoidea* | Alkaloids, Flavonoids, Terpenoids | Nyingchi, Xizang | Fu, J. J. (2020). Analysis of Alkaloids, Nutrients, and Toxicity Evaluation of Four *Ligularia* Species. Northwest A&F University. |
| *Anaphalis lactea Maxim.* | Organic acids, Others | Golog, Garzê | Liu, H. J., Zong, R. X., Liu, J. P., et al. (2018). Effects of Grassland Community Types on Population Characteristics, Morphology, and Resistance of *Anaphalis lactea* Male and Female Plants. Acta Prataculturae Sinica, 27(10), 113–124. |
| *Ajania tenuifolia* | Terpenoids, Lipids and lipid-like molecules, Flavonoids | Gansu, Xizang, Sichuan, Qinghai | Zhang, B. Y. (2014). Analysis of the Composition of Volatile Oil of *Artemisia annua* and *Ajania tenuifolia* in Yanchiwan Natural Reserve. Gansu Agricultural University. 2013  Lu, D. D. (2018). Optimization of Total Flavonoids Extraction of Ajania Tenuifolia Responsesurfacemethod. Chemical Engineering Design Communications, 2018,44(03):165. |
| *Aster alpinus* | Terpenoids, Flavonoids | Xizang, Sichuan, Yunnan | Ma, H. P., He, L., Wang, X., Yang, F. F., Xi, H. R., & He, X. Y. (2016). Protective Effect of Total Flavonoids from *Aster Souliei Franch* on Mice under Simulated High Altitude Hypoxia Environment Medical. Journal of Chinese People's Liberation Army,2016, 28(06), 1–4. |
| *Aster albescens* | Others | Deqin | He, L., Cheng, D. L., & Pan, X. (1996). A Study on the Chemical Constituents of *Aster albscens Hand*,-Mazz. China Journal of Chinese Materia Medica, 1996, (08), 483–484+511. |
| *Rheum tanguticum* | Phenols, Carbohydrates | Gansu, Qinghai | Yang, J., Xie, X. L., Hu, Y. P., Wang, L., & Li, Y. (2009). Effects of *Rheum* tanguticum Extracts on Seed Germination and Seedling Growth of Wheat and *Elymus nutans* . Bulletin of Botanical Research, 2009, 29(3), 320–324. |
| *Polygonum viviparum* | Flavonoids, Phenols | Xizang | Wang, Q. (2022). Studies on chemical constituents and antioxidant ofthe different parts from *Polygonum viviparum*. Guangdong Pharmaceutical University. 2022.  Wei, X. J., Du, J., Wang, W., & Pei, S. Q. (2022). Effect of Different Treatments on Seed Germination of *Polygonum viviparum* in Alpine Areas. Journal of Grassland and Forage Science, 2002, (02), 28–35. |
| *Rumex nepalensis* | Terpenoids, Lipids and lipid-like molecules, Phenols, Flavonoids | Southern Gansu, Southwestern Qinghai, Yunnan, Xizang, Sichuan | Wang, N., Zhu, B., Sheng, H. F., Chen, Y., & Yang, Y. (2011). Chemical Constituents of *Rumex nepalensis*. China Journal of Experimental Traditional Medical Formulae, 2011, 17(19), 132–136. |
| *Pedicularis kansuensis* | Terpenoids, Flavonoids, Alkaloids, Organic nitrogen compounds | Gansu, Qinghai, Sichuan, Xizang, Nujiang, Lijiang | Hou, Y., Cheng, X. Y., Ren, G. H., Deng, B., & Shang, Z. H. (2011). Allelopathic Effects of the Typical "Black Soil Land" Poisonous Plant on *Pedicularis kansuensis* in Qinghai-Tibetan Plateau. Acta Botanica Boreali-Occidentalia Sinica, 2011,31(08):1651-1656.  Bao, G. S., & Wang, H. S. (2011). Allelopathic Effects of *Pedicularis kansuensis Maxim*. on Several Graminaceous Grass Species on Alpine Meadow. Chinese Journal of Grassland, 2011,31(8), 1651–1656. |
| *Chamerion angustifolium* | Phenols, Flavonoids, Fatty acids, Others | Qinghai, Western Sichuan, Northwestern Yunnan, Xizang | Han, Y. Y., Liao, C. S., Tang, C., & Tang, J. G. (2023). Research Progress on Chemical Constituents and Pharmacological Effects of *Epilobium angustifolium L*. Journal of Anhui Agricultural Sciences,51(15), 1–6. |
| *Gentiana straminea* | Terpenoids | Qinghai | Wang, Y., Nie, Y. J., Wang, Z. B., Yao, Z., Wang, M., & Lin, P. C. (2019). Content Determination of Active Ingredients in the Aerial Parts of Gentiana straminea Maxim in Different Regions of Qinghai Province. Journal of Natural Science of Hunan Normal University,2019, 42(01), 49–54. |
| *Buddleja asiatica* | Lignans, Phenols | Xizang, Sichuan | Xiao, D., Xu, H. N., Wang, H. X., Chen, L., Xie, H. Q., Ding, Z. X., Zuo, A. X., & Jiang, Z. Y. (2024). Chemical constituents from *Buddleja asiatica*. Chinese Traditional and Herbal Drugs,2024, 55(01), 38–42. |
| *Buddleja macrostachya var. griffithii* | Flavonoids | Qinghai, Xizang | Ouyang, S., Qin, S. S., Chen, W. F., Yan, Y. J., & Chen, Y. H. (2021). Prediction of potential distribution of Buddleja based on the Maxent and ARCGIS. Ecological Science, 2021, 40(03):165-173. |
| *Aconitum flavum* | Alkaloids | Qinghai, Xizang, Sichuan | Li, H. L. (2012). Allelopathic Effects of *Aconitum flavum Hand-Mazz* on Several Graminaceous Grass Species on Alpine Meadow. Journal of Grassland and Forage Science. 2012, 34(3), 20–26.  Zhou, M. C. (2013). Allelopathic Effects of *Aconitum flavum Hand-Mazz* on Several Graminaceous Grass Species on Alpine Meadow. China Herbivore Science. 2013, 35(2), 31–36. |
| *Gymnaconitum gymnandrum* | Alkaloids | Xizang, Western Sichuan, Qinghai, Southern Gansu | Cao, L. (2013). Studies on breaking seed dormancy and the allelopathy of *Aconitum gymnandrum Maxim*. Gansu Agricultural University. 2013 |
| *Aconitum pendulum* | Flavonoids, Terpenoids | Xizang, Northwestern Yunnan, Western Sichuan, Qinghai, Southern Gansu | Liang, J. (2020). Allelopathic Potential of Major Poisonous Weeds andGrasses in Alpine Meadows. Qinghai University. |
| *Anemone obtusiloba* | Terpenoids, Alkaloids | Xizang, Sichuan | Lan, W. L. (2016). The research of allelopathy in *Anemone obtusiloba*. Lanzhou University of Technology.2016  Lv, W. L., Liu, Z. J., Zhang, X. R., & Wang, Y. X. (2018). Research of *Anemone obtusiloba* main pigment composition and mechanism of adaptation. Natural Product Research and Development,2019,31(03):395-400. |
| *Argentina lineata* | Flavonoids | Lijiang, Shangri-La | Chen, X. (2015). Compoent Analysis and Property Study of Flavonoids from *Potentilla fulgens*. Journal of Tianjin University of Commerce,2014, 37(6), 60.  Chen, X., Jiang, Z. T., & Li, R. (2013). Extraction of flavonoids from *Potentilla fulgens* by microwave method using ethanol as extractant and evaluation of antioxidant activity by cell model. Science and Technology of Food Industry, 2014,35(03):123-129. |
| *Potentilla acaulis* | Lipids and lipid-like molecules, Phenols | Qinghai | Wang, F. C., Dong, Q. M., Feng, B., Liu, Y. Z., Yu, Y., Yang, X. X., Zhang, C. P., Cao, Q., & Liu, W. T. (2023). Effects of single and mixed grazing of yak and Tibetan sheep on the balance betweenvegetative and reproductive growth of *Potentilla acaulis* in alpine grassland. Pratacultural Science, 2023, 40(07), 1866–1874.  Ren, X. Z., Guo, H. R., Ge, Y., Zhang, Y. L., & Wang, K. (2010). Allelopathy of Leaf-stem and Root Aqueous Extracts from *Potentilla acaulis Linn*. Chinese Journal of Grassland, 2010, 32(05), 51–56. |
| *Potentilla anserina* | Flavonoids | Xizang, Qinghai | Yan, Y. Y., Zhang, C., & Zhang, W. H. (2023). Study on composition identification and antioxidant activity of flavonoids of Tibet *Potentilla anserina L*. Journal of Food Safety & Quality, 2023,14(20):86-95. |
| *Ｐotentilla nivea* | Flavonoids, Terpenoids | Xizang | Gao, Y. P., Zhang, Y. M., & Liu, H. (2022). Chemical constituents from *Potentilla nivea*. Chinese Traditional Patent Medicine, 2022, 44(02), 456–460. |
| *Morina chinensis* | Terpenoids | Southern Qinghai, Western Sichuan | Quan, X. L. (2022). Allelopathic potential of cultivated grasses and main weeds in sowngrassland of alpine regions. Qinghai University. 2022. |
| *Stellera chamaejasme* | Shikimates and phenylpropanoids, Flavonoids, Lignans | Shangri-La, Xizang, Gansu | Cheng, J. N. (2022). Effects of the rhizosphere microenvironmentof *Stellera chamaejasme* L. on its population. Journal of Lanzhou University,2022.  Qin, B. (2013). Allelopathic Effects of *Stellera chamaejasme*. China Conference on Ecological Research, 2013, 35, 33. |
| *Cyperus rotundus* | Terpenoids | Yunnan, Sichuan, Southern Gansu | Zhang, J. X., & Zhao, B. S. (1996). Effect of seed treatment with extract B from Cyp erus rotundus. Chinese Journal of Applied Ecology,1996,7(2),218–220.  Xu, Z. M., Li, Z. Y., Zhu, D. J., Li, Y., Wang, X. X., & Li, Y. (2017). Preliminary Study on the Allelopathy of Weeds with *Medicago sativa*. Journal of Grassland and Forage Science, 2017, 37(6), 61–64, 78. |
| *Silene bilingua* | Lipids and lipid-like molecules, Terpenoids, Flavonoids, Alkaloids | Deqin, Nujiang, Southeastern Xizang, Sichuan | Qiao, D. (2018).Taxonomic Study of The Genus Silene (Caryophyllaceae) in Hengduan Mountains and Yunnan Plateau. Yunnan University. 2018 |
| *Pedicularis rex* | Shikimates and phenylpropanoids, Terpenoids | Southwestern Sichuan, Northwestern Yunnan | Chu, H. B., Zhang, Z. H., Chen, D., & Wang, Q. (2016). Determination of Phenylpropanoids and Anti-fatigueActivity of Effective Fractions of *Pedicularis rex*. Natural Product Research and Development, 2016,28(05):754-760. |
| *Verbascum thapsus* | Shikimates and phenylpropanoids, Terpenoids, Flavonoids | Xizang, Yunnan, Sichuan | Han, L. J., Gong, J., Luo, R. F., Zhao, T., Ni, S. F., Gao, A., Luo, B. B., & Zhang, M. (2010). Overview of Pharmaceutical Research on *Verbascum L. spp*. Journal of Anhui Agricultural Sciences, 2010,38(26):14346-14347. |
| *Cuscuta chinensis* | Flavonoids | Gansu, Sichuan, Yunnan | Huang, H. L., Cao, S. N., Pang, J., Wu, X. H., Luo, Q. C., Wu, Y. W., & Liu, D. J. (2024). Allelopathic Effects of Invasive Plants *Mikania micrantha* and *Cuscuta chinensis* on Eucalyptus spp. Eucalypt Science & Technology. 2024,41(03):52-58. |
| *Salix cupularis* | Alkaloids | Gansu, Qinghai, Sichuan | Zhao, Y. S., Yang, H., Xu, C. L., Jing, Y. Y., Li, Y., & Yu, X. J. (2023). Allelopathic Effects of water extracts from Salix cerebralis on seed germination of six grassland plant species in alpine shrub grassland. Grassland and Turf, 2023,43(02):67-74. |
| *Corydalis adunca* | Alkaloids | Deqin | Feng, H. T., Liu, X., Ye, B. G., et al. (2024). Studies on the Alkaloids From *Corydalis Adunca Maxim*. Asia-Pacific Traditional Medicine,2024, 20(05), 58–61. |
| *Iris bulleyana* | Flavonoids, Terpenoids, Benzenoids, Shikimates and phenylpropanoids | Shangri-La | He, Y. F., He, G. W., Liu, L. X., Wang, F., He, J. H., Chen, X. L., Du, J., & Guo, A. W. (2022). Analysis on chemical components in above-ground part of *Iris bulleyana* from Shangri-La of Yunnan Province. Journal of Plant Resources and Environment, 2022, 30(02), 22–38. |
| *Cynoglossum amabile* | Alkaloids | Xizang, Sichuan, Yunnan, Southern Gansu | Fan, Q. Y., Zhang, H. H., & Wang, W. H. (2016). Research on Separation and Purification of Alkaloids in C*ynoglossum amabile Stapf et Drumm* with Resin. Journal of Instrumental Analysis. 2016, 35(10), 1338–1342.  Fan, Q. Y., Xu, W. J., & Dai, C. M.(2019). Determination of Eight Alkaloids in *Cynoglossum amabile Stapf et Drumm* by Ultrahigh Performance Liquid Chromatography-Tandem Mass Spectrometry with Aqueous Two-phase Extraction. Journal of Instrumental Analysis. 2019, 38(11), 1328–1334. |

**Table S2. Model performance statistics for 83 native invasive plant species on the Qinghai-Tibet Plateau.**

| **Species** | **Training samples** | **Mean training AUC** | **Test samples** | **Mean test AUC** | **Test AUC standard deviation** |
| --- | --- | --- | --- | --- | --- |
| *Aconitum flavum* | 72 | 0.9877 | 24 | 0.9873 | 0.0022 |
| *Aconitum pendulum* | 213 | 0.9796 | 71 | 0.9786 | 0.002 |
| *Ajania tenuifolia* | 95 | 0.9892 | 31 | 0.9874 | 0.0018 |
| *Ajuga lupulina* | 215 | 0.9797 | 71 | 0.979 | 0.0023 |
| *Anaphalis lactea* | 89 | 0.9892 | 29 | 0.9863 | 0.0023 |
| *Anemone obtusiloba* | 51 | 0.9912 | 16 | 0.9889 | 0.0021 |
| *Anisodus tanguticus* | 233 | 0.9765 | 77 | 0.9771 | 0.0022 |
| *Argentina lineata* | 72 | 0.9879 | 24 | 0.9879 | 0.0022 |
| *Artemisia nanschanica* | 323 | 0.9654 | 107 | 0.9647 | 0.0026 |
| *Aster albescens* | 7 | 0.9984 | 2 | 0.9947 | 0.0022 |
| *Aster alpinus* | 57 | 0.9938 | 18 | 0.9917 | 0.0019 |
| *Astilbe rivularis* | 36 | 0.9944 | 12 | 0.989 | 0.0028 |
| *Astragalus yunnanensis* | 224 | 0.9764 | 74 | 0.9762 | 0.0021 |
| *Buddleja asiatica* | 34 | 0.996 | 11 | 0.9926 | 0.0019 |
| *Buddleja macrostachya var. griffithii* | 52 | 0.9938 | 17 | 0.9937 | 0.0016 |
| *Chamerion angustifolium* | 210 | 0.9794 | 69 | 0.9763 | 0.0036 |
| *Chelonopsis souliei* | 9 | 0.9954 | 3 | 0.9776 | 0.012 |
| *Clinopodium repens* | 59 | 0.9928 | 19 | 0.9914 | 0.0019 |
| *Corydalis adunca* | 69 | 0.9926 | 23 | 0.9908 | 0.0017 |
| *Cuscuta chinensis* | 37 | 0.9951 | 12 | 0.9942 | 0.0015 |
| *Cynoglossum amabile* | 61 | 0.9914 | 20 | 0.9916 | 0.0023 |
| *Cyperus rotundus* | 5 | 0.9972 | 1 | 0.9939 | 0.0002 |
| *Elsholtzia densa* | 369 | 0.967 | 122 | 0.9631 | 0.0028 |
| *Elymus nutans* | 374 | 0.9617 | 124 | 0.958 | 0.0031 |
| *Erigeron multiradiatus* | 150 | 0.984 | 50 | 0.9843 | 0.0018 |
| *Euphorbia jolkinii* | 27 | 0.997 | 8 | 0.9957 | 0.0014 |
| *Festuca coelestis* | 195 | 0.978 | 65 | 0.9781 | 0.0023 |
| *Galeopsis bifida* | 205 | 0.9812 | 68 | 0.9799 | 0.002 |
| *Gentiana straminea* | 222 | 0.9777 | 74 | 0.9764 | 0.0022 |
| *Gymnaconitum gymnandrum* | 144 | 0.9847 | 47 | 0.9838 | 0.002 |
| *Hippophae gyantsensis* | 25 | 0.996 | 8 | 0.9928 | 0.0028 |
| *Hippophae rhamnoides subsp. yunnanensis* | 150 | 0.9846 | 49 | 0.9834 | 0.0021 |
| *Hippophae tibetana* | 380 | 0.9635 | 126 | 0.9633 | 0.0025 |
| *Hyoscyamus niger* | 136 | 0.9856 | 45 | 0.9846 | 0.0019 |
| *Iris bulleyana* | 48 | 0.9934 | 16 | 0.9921 | 0.0017 |
| *Isodon wardii* | 18 | 0.9908 | 6 | 0.9901 | 0.0041 |
| *Leontopodium nanum* | 248 | 0.9743 | 82 | 0.9699 | 0.0037 |
| *Leontopodium souliei* | 209 | 0.9785 | 69 | 0.9782 | 0.0021 |
| *Leucosceptrum canum* | 40 | 0.9931 | 13 | 0.9881 | 0.0042 |
| *Ligularia caloxantha* | 8 | 0.999 | 2 | 0.9981 | 0.0006 |
| *Ligularia cymbulifera* | 23 | 0.9981 | 7 | 0.9971 | 0.0008 |
| *Ligularia dictyoneura* | 28 | 0.9966 | 9 | 0.9965 | 0.0009 |
| *Ligularia discoidea* | 8 | 0.9988 | 2 | 0.9986 | 0.0006 |
| *Ligularia duciformis* | 33 | 0.9962 | 10 | 0.9947 | 0.0012 |
| *Ligularia hodgsonii* | 43 | 0.993 | 14 | 0.9921 | 0.0017 |
| *Ligularia kanaitzensis* | 14 | 0.9985 | 4 | 0.9981 | 0.0007 |
| *Ligularia lamarum* | 32 | 0.9942 | 10 | 0.9919 | 0.002 |
| *Ligularia lapathifolia* | 18 | 0.9952 | 6 | 0.9902 | 0.0049 |
| *Ligularia lidjiangensis* | 8 | 0.9992 | 2 | 0.9988 | 0.0005 |
| *Ligularia × maoniushanensis* | 6 | 0.9942 | 2 | 0.9956 | 0.0015 |
| *Ligularia nelumbifolia* | 26 | 0.9971 | 8 | 0.9959 | 0.0011 |
| *Ligularia paradoxa* | 9 | 0.9875 | 2 | 0.961 | 0.024 |
| *Ligularia sagitta* | 42 | 0.9958 | 13 | 0.9958 | 0.0009 |
| *Ligularia stenocephala* | 18 | 0.9985 | 5 | 0.9981 | 0.0006 |
| *Ligularia tongolensis* | 75 | 0.9923 | 24 | 0.9919 | 0.0015 |
| *Ligularia veitchiana* | 18 | 0.9925 | 5 | 0.934 | 0.0568 |
| *Ligularia vellerea* | 33 | 0.997 | 10 | 0.9958 | 0.0012 |
| *Ligularia virgaurea* | 233 | 0.9793 | 77 | 0.9787 | 0.002 |
| *Morina chinensis* | 110 | 0.9879 | 36 | 0.9886 | 0.0017 |
| *Morina kokonorica* | 167 | 0.9813 | 55 | 0.979 | 0.0024 |
| *Nepeta hemsleyana* | 57 | 0.9911 | 18 | 0.9887 | 0.0038 |
| *Origanum vulgare* | 117 | 0.9865 | 39 | 0.985 | 0.0022 |
| *Oxytropis glabra* | 63 | 0.9911 | 21 | 0.9907 | 0.0021 |
| *Oxytropis ochrocephala* | 33 | 0.9917 | 11 | 0.9737 | 0.0201 |
| *Pedicularis kansuensis* | 282 | 0.9714 | 93 | 0.9667 | 0.0043 |
| *Pedicularis rex* | 13 | 0.9977 | 4 | 0.9968 | 0.0013 |
| *Peganum harmala* | 6 | 0.9996 | 2 | 0.9993 | 0.0003 |
| *Peganum multisectum* | 26 | 0.9968 | 8 | 0.9964 | 0.0017 |
| *Phlomoides rotata* | 294 | 0.9738 | 97 | 0.9727 | 0.0022 |
| *Polygonum viviparum* | 193 | 0.9773 | 64 | 0.9764 | 0.0025 |
| *Potentilla acaulis* | 21 | 0.9978 | 6 | 0.9971 | 0.001 |
| *Potentilla anserina* | 81 | 0.9919 | 26 | 0.9884 | 0.0023 |
| *Potentilla nivea* | 287 | 0.8821 | 95 | 0.8622 | 0.0171 |
| *Rheum tanguticum* | 42 | 0.9952 | 13 | 0.9936 | 0.0019 |
| *Rumex nepalensis* | 160 | 0.9838 | 53 | 0.9831 | 0.0021 |
| *Salix cupularis* | 12 | 0.9964 | 4 | 0.9896 | 0.0055 |
| *Saussurea pulchra* | 10 | 0.9975 | 3 | 0.9963 | 0.0017 |
| *Silene bilingua* | 24 | 0.9975 | 8 | 0.9969 | 0.0008 |
| *Sophora alopecuroides* | 125 | 0.986 | 41 | 0.9854 | 0.0023 |
| *Stellera chamaejasme* | 39 | 0.9938 | 13 | 0.9919 | 0.0017 |
| *Thermopsis lanceolata* | 287 | 0.9724 | 95 | 0.9713 | 0.0024 |
| *Tibetia yunnanensis* | 73 | 0.9921 | 24 | 0.9918 | 0.0015 |
| *Verbascum thapsus* | 657 | 0.924 | 218 | 0.9205 | 0.0065 |

**Note:** The table presents detailed metrics from MaxEnt, including the number of training and test samples, mean training AUC, mean test AUC, and test AUC standard deviation. All models were developed using 10 bootstrap replicates with a 75%/25% training-test data split. The models demonstrated excellent predictive accuracy, with overall mean training AUC of 0.9867 and mean test AUC of 0.9834 across all species. All test AUC standard deviation values were below 0.06, indicating high model stability across bootstrap iterations. Species are listed alphabetically for reference.

**Table S3. Results of univariate Ordinary Least Squares (OLS) and Spatial Autoregressive Model (SAR) analyses on the factors influencing the richness of native invasive plant species in the Qinghai-Tibet Plateau.**

|  | OLS | | SAR | |
| --- | --- | --- | --- | --- |
|  | Coef | R^2^ | Coef | R^2^ |
| SR: single variable of OLS and SAR | | | | |
| Bio18 | 0.036158*** | 0.4274 | -0.0030594 | 0.189 |
| Road | 0.029734*** | 0.3841 | -0.00042091 | 0.197 |
| Bio3 | 0.82656*** | 0.1752 | 0.88787*** | 0.234 |
| GDP | 7.990e-04*** | 0.09024 | -5.6774e-05 | 0.241 |
| Population | 3.656e-05*** | 0.07908 | -1.3516e-05 | 0.242 |
| Allelochemicals | 1.6676*** | 0.05228 | -0.044063 | 0.156 |
| Bio15 | -0.09125*** | 0.03001 | 0.023795 | 0.248 |

**Note:** R^2^ (or pseudo R^2^) and regression coefficients (Coef) are provided. *P<0.05, **P<0.01, ***P< 0.001.

**Table S4. Moran's I index of predictors explaining native invasive plant distribution in the Qinghai-Tibet Plateau.**

| Variables | Moran I statistic | p-value |
| --- | --- | --- |
| Bio18 | 0.9043064862 | *** |
| Road | 0.9474317027 | *** |
| Bio3 | 0.9823687391 | *** |
| GDP | 0.800317653 | *** |
| Population | 0.9425401214 | *** |
| Allelochemicals | 0.7988368872 | *** |
| Bio15 | 0.9532272593 | *** |

**Note:** Moran's I value close to 1 indicates positive spatial autocorrelation in the data, meaning features with similar attribute values tend to be clustered in space. When Moran's I approaches 0, it suggests that the data are randomly distributed in space, showing no statistically significant spatial autocorrelation pattern. *P<0.05, **P<0.01, ***P< 0.001.

**Table S5. Future expansion of invasive species hotspots and its overlap with protected areas (PAs) on the Qinghai-Tibet Plateau (QTP).**

| **Scenario** | **Period** | **Area (10⁴ km²)** | | **Percentage of Total Area Covered (%)** | |
| --- | --- | --- | --- | --- | --- |
|  | | Expanded Habitat | Overlap with PAs | QTP | PAs |
| SSP1-2.6 | 2021-2040 | 199.26 | 46.31 | 78.56 | 74.88 |
|  | 2081-2100 | 197.04 | 44.56 | 77.68 | 72.04 |
| SSP5-8.5 | 2021-2040 | 197.62 | 46.00 | 77.91 | 74.38 |
|  | 2081-2100 | 121.29 | 14.58 | 47.82 | 23.57 |

**Notes:**

**Abbreviations:** QTP, Qinghai-Tibet Plateau; PAs, Protected Areas.

**Constant areas:** The total area of the QTP (253.65 × 10⁴ km²) and PAs (61.85 × 10⁴ km²) were calculated directly from raster data under the Asia_North_Albers_Equal_Area_Conic projection to preserve areal accuracy.

**Data presentation:** Model projections are based on the BCC-CSM2-MR global climate model (GCM). The term 'Expanded Habitat' refers to the newly suitable area for invasive species hotspots under future climate scenarios, as defined against the current baseline period. Both area and percentage values are rounded to two decimal places.

**Calculation of percentages:** '% of QTP Area Covered' was calculated as (Expanded Habitat Area / 253.6 × 10⁴ km²) × 100%. '% of PA Area Covered' was calculated as (Overlap Area with PAs / 61.8 × 10⁴ km²) × 100%.

**FIGURE S1.** **Human factors and the distribution patterns of native invasive plant species richness on the Qinghai-Tibet Plateau.**


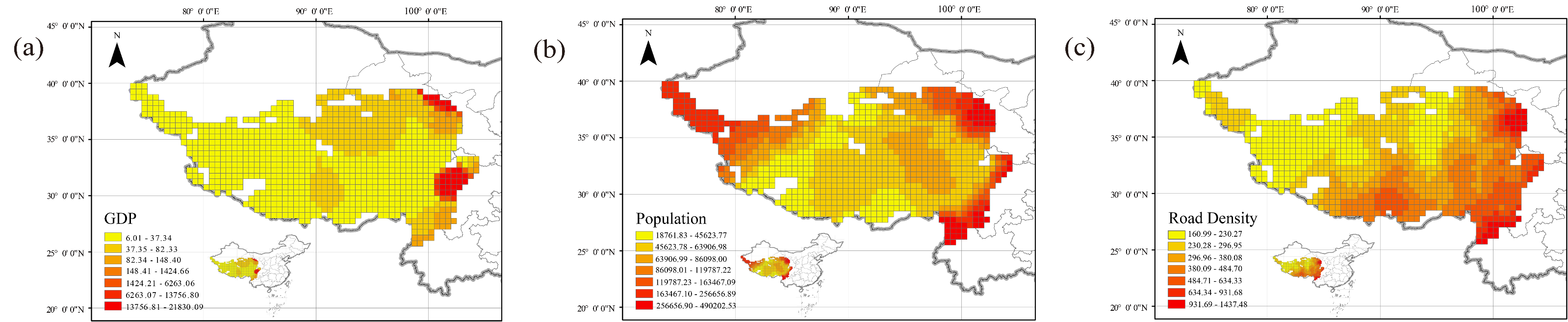


**FIGURE S2.** **Climate factors and the distribution patterns of native invasive plant species richness on the Qinghai-Tibet Plateau.**

**
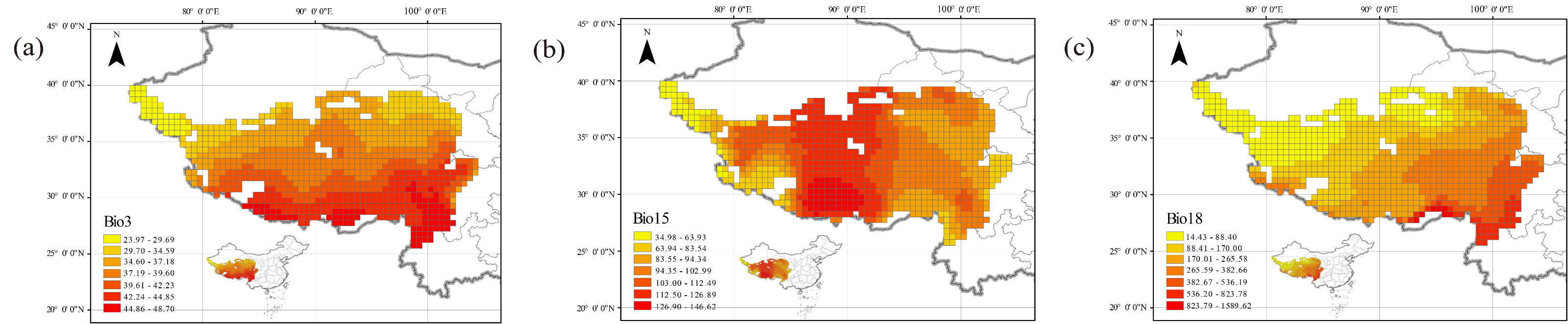
**

**Note:** Bio3: isothermality, Bio15: precipitation seasonality, Bio18: precipitation of warmest quarter.

**FIGURE S3. The first seven important factors in the Bayesian model were standardized to a scale range from -2.5 to 7.5.**


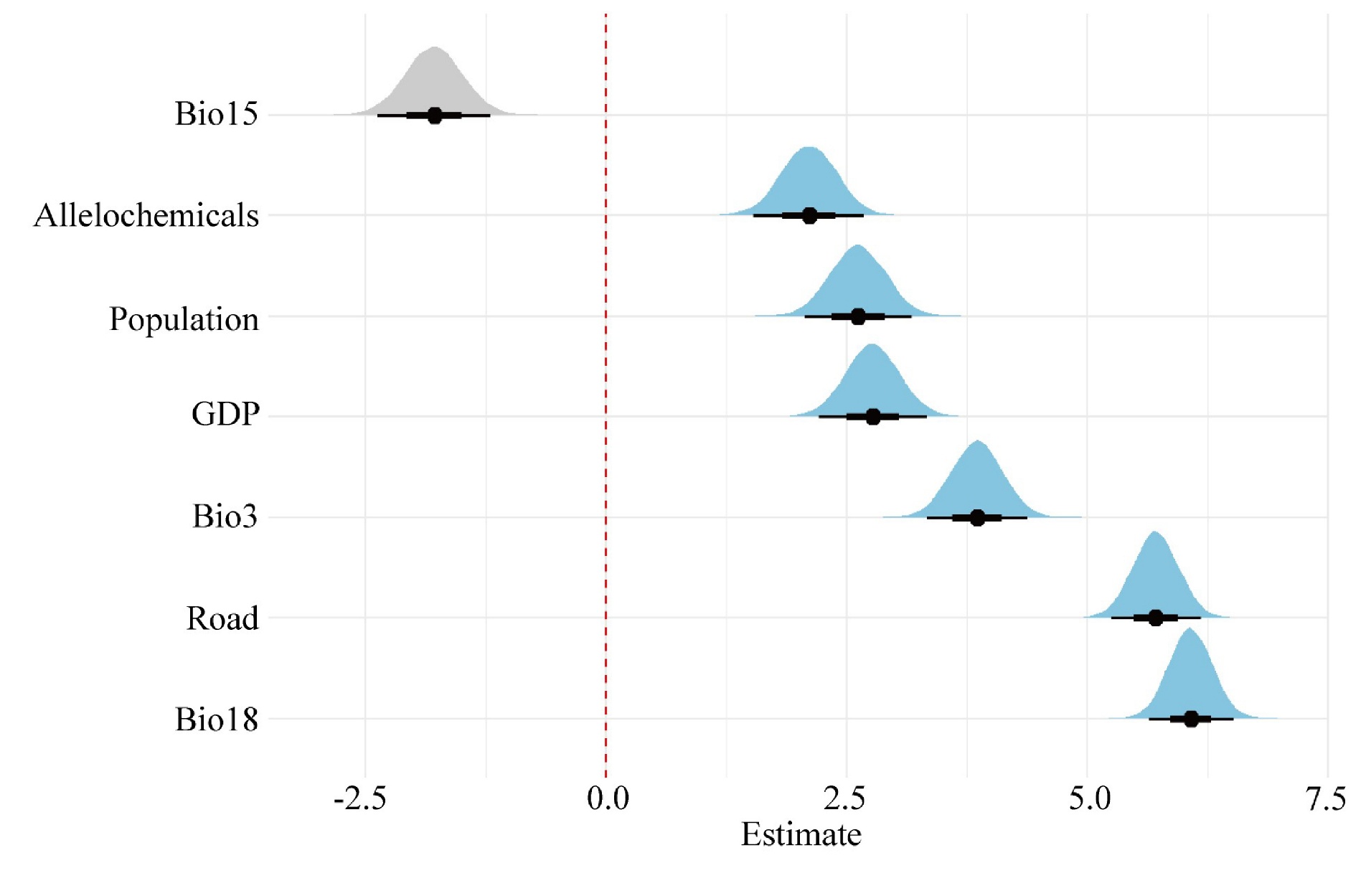

Supplement: Supplementary file 1 [file DataSheet1.docx]
